# An archaeobotanical and stable isotope approach to changing agricultural practices in the NW Mediterranean region around 4000 BC

**DOI:** 10.1177/09596836231211848

**Published:** 2023-12-04

**Authors:** Ferran Antolín, Stefanie Jacomet, Raül Soteras, Claudia Gerling, Stefano M Bernasconi, Franziska Follmann, Irka Hajdas, Madalina Jaggi, Ana Jesus, Héctor Martínez-Grau, Francesc Xavier Oms, Brigitte Röder, Bigna L Steiner, Samuel van Willigen

**Affiliations:** 1 German Archaeological Institute, Natural Sciences Unit, Germany; 2 Department of Environmental Sciences, Integrative Prehistory and Archaeological Science (IPAS), Basel University, Switzerland; 3 Departement Altertumswissenschaften, Ur- und Frühgeschichtliche und Provinzialrömische Archäologie, Basel University, Switzerland; 4 Department of Earth Sciences, Geological Institute, ETH Zurich, Switzerland; 5 Institut für Prähistorische Archäologie, Freie Universität Berlin, Germany; 6 Laboratory of Ion Beam Physics (LIP), Swiss Federal Institute of Technology (ETH) Zurich, Switzerland; 7 Seminari d’Estudis i Recerques Prehistòriques (SERP). Secció de Prehistòria i Arqueologia, Dept. d’Història i Arqueologia, Facultat de Geografia i Història. Universitat de Barcelona, Spain; 8 Institut d’Arqueologia de la Universitat de Barcelona. Facultat de Geografia i Història. Universitat de Barcelona, Spain; 9 InSitu Archéologie, Switzerland

**Keywords:** carbon, nitrogen, radiocarbon dating, prehistoric agriculture, Neolithic, France, Spain

## Abstract

It has recently been observed, that a change in the crop spectrum happened during the so-called Middle Neolithic in France at ca. 4000 BC. An agricultural system based on free-threshing cereals (naked wheat and naked barley) seems to shift to one based on glume wheats. This is a major change for traditional farmers and this paper aims to shed light on its possible causes. Here we describe the results of new investigations in a key area for the understanding of this process: the NW Mediterranean arch, where free-threshing cereals are the main cultivars since ca. 5100 BC. New data confirm that the shift towards glume wheats is also observed in some sites of the NE of the Iberian Peninsula and that among the glume wheats that spread at ca. 4000 BC we should not only consider emmer and einkorn but also Timopheevi’s wheat. Stable isotope analyses indicate no major decrease in soil fertility or alterations in local precipitation regimes. The agricultural change may be the result of a combination of the spread of damaging pests for free-threshing cereals and presumably new networks being developed with the North-eastern part of Italy and the Balkans.

## Introduction

Until a decade ago, the period between 4500 and 3700/3500 BC was seen as a phase of relative stability in Southern France and its surrounding regions (Northern Italy, NE Spain and Switzerland). This time period was connected to the so-called Middle Neolithic cultures of ‘Chassey’ (in France), ‘Sepulcres de Fossa’ (in Catalonia, NE of Spain) and ‘Vasi a Bocca Quadrata’ (VBQ) (in Northern Italy) and ‘Cortaillod’ (in Western Switzerland). In Southern France and NE Spain, this phase is associated with higher site density, larger open air sites, ‘fully-developed’ farming economies, associated burial areas, large exchange networks of prestige objects, etc. (e.g. Antolín et al., 2015; Beeching et al., 1991; Berger et al., 2019; Borrello and Van Willigen, 2012; Bréhard et al., 2010; Clop García, 2010; Delhon et al., 2009; Martin et al., 2016; Molist, 1992; Mottes, 2021; Terradas and Gibaja, 2002; Vaquer and Lea, 2011). These cultures ended up defining the Middle Neolithic as a whole, homogenising multiple dynamics of local or regional scale and integrating them in either the phase of formation, development or disintegration of the core Middle Neolithic culture. Van Willigen has analysed this period with detail and argues that most of the narrative has been generated from sites dated to the fourth millennium BC and there has been a lack of information for the second half of the fifth millennium BC (van Willigen, 2020). In general, this period can be considered poorly dated, since most of the available dates belong to funerary contexts (Martínez-Grau et al., 2021) and hence do not necessarily date socio-economic dynamics. Recent syntheses (Martin et al., 2016) and data generated by newly investigated sites such as Les Bagnoles (Isle-sur-la Sorgue, Vaucluse) (van Willigen et al., 2020b) or Isolino Virginia (Lake Varese, Lombardy) (Antolín et al., 2022, in press; Steiner et al., in press) are contributing to change the picture of the period. In connection to these sites, the project AgriChange (Antolín et al., 2018a), funded by the Swiss National Science foundation (2018–2022), has performed multiproxy analyses to better reconstruct agricultural dynamics during the Neolithic in the area.

One of the most significant changes in the paradigm around the middle Neolithic is that there was an important crop change around 4000 BC, from free-threshing cereals (naked wheat and naked barley) to glume wheats (einkorn and emmer), observed for the French territory (Martin et al., 2016). A similar change had been observed in other areas, such as Switzerland, happening much later in time, ca. 3500 BC (Jacomet, 2006, 2007). The archaeobotanical dataset for the fifth millennium BC is relatively scanty, especially in Southern France and particularly for the second half of the fifth millennium cal BC. Previous work on this period in Northern Italy (Carra, 2013) and Catalonia (Antolín et al., 2015) had failed to identify this change of trend due to a poor chronological accuracy and the lower number of investigated sites in these areas in comparison to France. During the fifth millennium BC, free-threshing cereals – either naked wheat or barley or both – are clearly dominating in Catalonia (Antolín et al., 2018b; Antolín and Schäfer, 2020), Southern France (Martin et al., 2016; van Willigen et al., 2020a) and Northern Italy (Antolín et al., in press; Steiner et al., in press). The switch from free-threshing cereals to glume wheat is a relevant one for small-scale farmers, since it means changing the agricultural system completely: glume wheats are usually stored in spikelets, dehusked on a daily basis and often consumed differently (e.g. as bulgur) while free-threshing cereals are usually processed in bulk, stored as grain and more often transformed into flour or semolina (Antolin et al., 2016; Hillman, 1984; Pena-Chocarro et al., 2009). The study region is key to understand the process, since it seems to be the first region in Western Europe where free-threshing cereals became the dominant cultivars around 5100 BC (Antolín et al., 2015; Bouby et al., 2020a; de Vareilles et al., 2020).

Previous research has pointed out some of the potential reasons for this change: a deterioration of the climate, soil depletion or new cultural contacts (Jesus et al., 2021; Martin et al., 2016). Climatically, this time period coincides with the transition phase between the early (wetter) and the late (drier) Holocene in the area (i.e. Jalut et al., 2009; Magny et al., 2013), and some studies indicate a cold period between 4600 and 3750 BC (i.e. Jalali et al., 2016). These changes are difficult to quantify and correlate with archaeological evidence, but they might explain the temporary abandonment of the cereals that thrived during the Climatic Optimum in the area. From a cultural point of view, researchers observed changes in the funerary record and between pottery traditions that would go in parallel to the crop changes, such as between the Montboló and Sepulcres de Fossa cultures in Catalonia, the Chassey and the La Roberte in Provence (Southern France), or the Vasi a Bocca Quadrata and Lagozza in North-western Italy (Perrin et al., 2016; van Willigen, 2020). It is therefore possible, that these changes in agricultural practices actually indicate more profound socio-economic changes not yet fully understood. Another possibility was raised recently: the widespread presence of pests (Antolín et al., in press; Häberle et al., 2022). Indeed, in two of the case studies of the AgriChange project, both sites with waterlogged preservation conditions, evidence of pests (both insects and small animals) has been reported for the period around 4000 BC.

The goal of this paper is to approach this problematic around the crop change and its possible causes by integrating data from recently investigated sites in Southern France and Catalonia dated ca. between 4500 and 3700 BC, all of them with direct radiocarbon dates on seed remains and combining the archaeobotanical information with carbon and nitrogen stable isotope analysis. Stable isotope analysis from cereal remains inform us about the growing conditions of the plants (Fiorentino et al., 2015). Plants normally react to a reduction or increase in water availability through the closing or opening of their stomata. As indicators of water availability during tissue formation (Farquhar et al., 1989), carbon isotope values obtained from cereal grains offer reliable data on water availability for ancient crops (Araus et al., 1997a; Ferrio et al., 2005; Fiorentino et al., 2015). Other factors, such as altitude, latitude and temperature, further influence the carbon isotope values of plant tissues (Diefendorf et al., 2010; Körner et al., 1988). As there is no evidence of ancient artificial irrigation in Neolithic Europe, the carbon isotope compositions must be interpreted as a result of the interaction of the characteristics of the soil with precipitation during the grain-filling period. Nitrogen isotope ratios obtained from crop remains may be affected by several factors such as precipitation (Styring et al., 2017a), presenting enriched values when aridity increases. Otherwise, δ^15^N values are a reliable proxy for nutrient availability in the soils and can be interpreted in terms of soil quality or even manuring under the adequate circumstances (Bogaard et al., 2013; Styring et al., 2017a).

## Materials and methods

**Figure 1.**
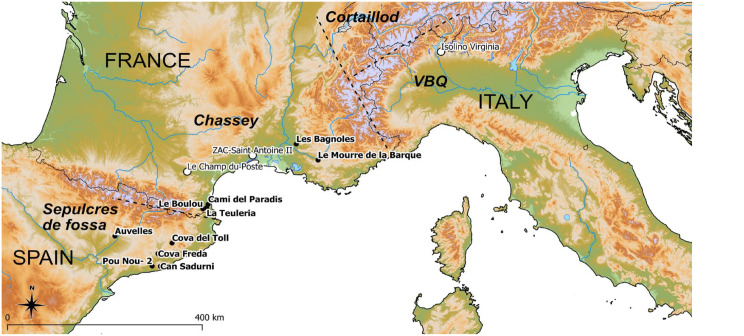
Location of investigated sites (black dots) and other sites mentioned in the text (white dots). Main technocultures are separated by dashed lines and written in italics (Software: QGIS3.6, © European Union, Copernicus Land Monitoring Service (2016), European Environment Agency (EEA)).

Data from 10 sites located between the NE of the Iberian Peninsula and the maritime Alps (Figure 1) has been generated and evaluated in the framework of this paper:

- Auvelles (Castelló de Farfanya, Lleida, Spain): open air site at ca. 355 m a.s.l. in the Pre-Pyrenees of Western Catalonia, with an annual precipitation that currently is around 400 mm. Numerous excavated structures (silo pits and other types of pits and hearths) were uncovered during the excavations in 2004. Ceramic technology indicated ‘Postcardial’ style and one radiocarbon date on naked barley allowed to date some features of the site around 4300–4100 BC (Table 1). Sediment samples were obtained but they were not processed until 2015. Charcoal analyses for the site were already published (Martín Seijo and PIqué i Huerta, 2008) and a monography is currently in press, where the seed and fruit remains will also be published (Antolín, in press). Most archaeobotanical remains come from one unit (88) with a presumable use as combustion feature or a refilling of a pit with material from a combustion feature.
- Cova del Toll (Moià, Barcelona, Spain): cave site at ca. 700 m a.s.l. and precipitation is around 250 mm per year. The samples analysed were recovered by J. Guilaine and colleagues in the 1970s and first analysed by Hopf (Guilaine et al., 1982). The data provided here belongs to the recent reidentification and quantification of the finds (Antolín, 2020). Most of the layers of this phase have been dated to the second half of the fifth millennium cal BC (c. 4500–3900 BC). During this period, this large cave was used at least as a pen and also as a storage place (Guilaine et al., 2020).
- Cova Freda (Collbató, Barcelona, Spain): cave site at ca. 500 m a.s.l. in the pre-litoral Catalan range, at 30 km of distance from the city of Barcelona, near the Llobregat river, and with a yearly precipitation of around 350 mm. It was first excavated ca. 100 years ago by Colomines and has recently been re-investigated by Oms and others (Oms et al., 2019). Middle Neolithic deposits connected to animal penning inside the cave were uncovered and radiocarbon dates of glume wheat grains confirmed their ascription to the period between 3950 and 3700 BC (Table 1). The investigations of the site are still on-going and only preliminary data will be presented here.
- Pou Nou-2 (Sant Pere Molanta, Barcelona, Spain): open air site at ca. 225 m a.s.l. in the pre-littoral plain in the central Catalan coast, where annual precipitations of ca. 550 mm are usually observed. Several negative features (pits) were documented and dated to several time periods between the Neolithic and the Medieval period. The bottom of a large pit (E3) with a burial was identified and excavated in 1993. Samples were taken and processed by the late Vicente López but the analysis was only performed in 2015 (involving new samples from the sediment around the burial that was kept in the museum) and published shortly after (Antolín et al., 2018b). The archaeobotanical remains belong to the deposits below the burial and it is possible that they are not part of the funerary episode. The radiocarbon dating of one naked wheat grain dates the deposit around 4200–4000 BC (Table 1). Most of the remains recovered in this feature corresponded to charred acorn cotyledons.
- Can Sadurní (Begues, Barcelona, Spain): cave site at ca. 425 m a.s.l. (with yearly precipitations of ca. 450 mm) at a few kilometres from the coast and the city of Barcelona, with Neolithic occupations between 5350 and 4000 BC. During the second half of the fifth millennium cal BC, the cave is used (alternatively) as a burial area (ca. 4400–4200 BC) and as a byre for sheep and goats (4800–4350 and probably also 4200–4000 BC) (Antolín et al., 2017; Bergadà et al., 2018; Edo et al., 2019; Edo and Antolín, 2016). Archaeobotanical presence/absence data was already published in a previous paper (Antolín and Schäfer, 2020) and the analyses of the samples taken until 2008 where part of a Master Thesis (Antolín, 2008), but we will focus on the penning deposits dated to 4500–4000 BC. The entirety of the sediment excavated after 2010 was processed with a flotation machine (more than 20,000 L of sediment), but only a selection of 5 squares (adding up to over 3000 of sediment) spread across the excavated area was analysed and quantified. Additionally, discrete burnt layers (mostly burnt dung deposits) were analysed completely. Within the chronology of this work we included two features, XIII and XIV (the remaining features fall within the period dated to 4800–4500 BC).
- Camí del Paradís (Corneilla-del-Vercol, Occitanie, France): open air site at ca. 10 m a.s.l. (with ca. 300 mm of yearly precipitation) excavated by Acter Archéologie. One could consider this coastal site a settlement of relatively short duration that dates to the period after 4000 BC. Some negative structures were found (pits and hearths).
- Le Cortal d’en Quircq (Le Boulou, Occitanie, France) – in this text referred to as Le Boulou: Open air site at 93 m a.s.l. excavated in 2019 by Acter Archéologie. A variety of excavated features (pits and hearts) was sampled, probably belonging to one large farm complex. Dates on naked barley and einkorn grains indicate that the site was occupied around 3900–3600 BC (Table 1). The average yearly precipitation at the site is around 305 mm.
- La Teuleria (Saint-Génis-des-Fontaines, Occitanie, France): open air site excavated in 2018 by Acter Archéologie located at 46 m a.s.l. and consisting of excavated features such as silos and other types of pits. A complete report of the excavation has been produced (Galin, 2020). Radiocarbon dates performed at the site suggest it was occupied ca. from 4200 to 3600 BC. The yearly precipitation at the site is ca. 475 mm.
- Les Bagnoles (Isle-sur-la-Sorgue, Western Provence, France): open air site located at 57 m a.s.l. with average yearly precipitation around 550 mm, on the ancient alluvial plain of the river Durance, with excavated features (pits, graves, wells and silo pits, among other features) mostly dating to the period between 4300 and 3700 BC, with two clear phases, one belonging to the Chassey pottery culture (4300–4000 BC) and one to La Roberte (3950–3700 BC). The archaeological context is fully published (van Willigen et al., 2020b) and it is to a certain point debated whether the first phase of the site had a ritual component and to which extent this could influence the archaeobotanical results stemming from the well deposits. In fact, archaeobotanical data from the wells (mostly found in an uncharred state) has already been published (Jesus et al., 2021) and suggests daily refuse (cereal chaff, edible fruits, locally growing natural vegetation, etc.). For this paper, we focus on the charred material and we hence include the unpublished analyses of the contents of the silo pit 1110 and the pit 449, which both date to the youngest occupation phase ca. 3950–3750 BC (Table 1).
- Le Mourre de la Barque (Jouques, Western Provence, France): small cave site, located at 300 m a.s.l., with a complex stratigraphy and diversity of uses, close to the river Durance. Yearly precipitations lie around 350 mm. It is excavated between 1993 and 2004. Stefanie Jacomet started the first archaeobotanical evaluations of the site, later continued by the AgriChange project. Neolithic occupations seem to stretch from the Early to the Late Neolithic, with probable hiatuses so far not precisely defined chronologically. We will focus on the Middle Neolithic deposits of the early fourth millennium BC -Layer 14CD- (van Willigen, 2010). The dating of a naked wheat grain resulted in the chronological period ca. 3770–3650 BC (Table 1).

**Table 1.** Radiocarbon dates of the investigated sites performed by the AgriChange Project.

Site	Sample_Code	Cultural_Phase	Layer	Sample	Species	Lab_Code	BP	SD	cal BC 2σ	δC13 (‰)	C/N	Publication
Auvelles	AU04_UN88_UE886_N24	Postcardial	886	Cereal	*Hordeum vulgare* var. *nudum*	ETH-96194	5388	28	−4336 to 4065	−25.1	19.02	Unpublished
Pou Nou-2	13_E3_NIIb_sectcentral	Postcardial	E3	Cereal	*Triticum aestivum/durum/turgidum*	ETH-107006	5236	24	−4221 to 3974	−20.8	20.94	Unpublished
Cova Freda	09_FRE_H12_IIb	Middle Neolithic	IIb	Cereal	*Triticum monococcum/dicoccum*	ETH-106985	5035	24	−3949 to 3715	−21.3	24.17	Unpublished
	06_FRE_H12_IIa	Middle Neolithic	IIa	Cereal	*Triticum monococcum*	ETH-106986	5024	23	−3946 to 3711	−22.3	26.54	Unpublished
Can Sadurní	12CS_E6_IIe_11	Postcardial	11	Cereal	*Hordeum vulgare* var. *nudum*	ETH-96196	5552	26	−4447 to 4347	−23.5	15.66	Unpublished
	13CS_E9_IIf_11	Postcardial	11	Cereal	*Hordeum vulgare* var. *nudum*	ETH-96199	5567	28	−4451 to 4350	−24.0	20.48	Unpublished
	13CS_INH2_IIf_11a3	Postcardial	11a3	Wild plant	*Arbutus unedo*	ETH-88894	5610	25	−4496 to 4359	−21.3	-	Edo et al. (2019)
	14CS_E6_IIg_11a4	Postcardial	11a4	Cereal	*Hordeum vulgare* var. *nudum*	ETH-96198	5707	29	−4656 to 4454	−24.0	21.69	Unpublished
Le Boulou	06_12_FS1447_US3013	Chassey	3013	Cereal	*Hordeum vulgare* var. *nudum*	ETH-106982	5012	23.5	−3943 to 3661	−21.6	43.83	Unpublished
	04_05_29_FS1493_US2619	Chassey	2619	Cereal	*Triticum monococcum*	ETH-106983	5089	23.7	−3961 to 3799	−18.5	40.23	Unpublished
Les Bagnoles	ISB15_622_994_P42_2	La Roberte II	994	Cereal	*Triticum monococcum*	ETH-88902	5027	26	−3947 to 3712	−16.3	36.72	Martínez-Grau et al. (2020)
	ISB15_567_1010_P11	La Roberte II	1010	Cereal	Cerealia (4 frgs)	ETH-88900	5036	25	−3950 to 3715	−24.4	369.61	Martínez-Grau et al. (2020)
	ISB15_512_St449_Pr10	La Roberte	St449	Cereal	*Triticum aestivum/durum/turgidum*	ETH-112990	5055	26	−3951 to 3786	−24.6	19.32	Unpublished
	994_p45	La Roberte II	994	Cereal	*Triticum* cf. *monococcum*	ETH-96173	5096	28	−3967 to 3799	−23.7	15.30	Martínez-Grau et al. (2020)
	ISB15_603_990_P25	La Roberte I	990	Cereal	*Triticum aestivum/durum/turgidum*	ETH-88904	5213	25	−4157 to 3965	−24.8	18.96	Martínez-Grau et al. (2020)
	ISB15_615_990_P70	La Roberte I	990	Cereal	*Triticum aestivum/durum/turgidum*	ETH-88901	5226	25	−4216 to 3969	−24.6	18.62	Martínez-Grau et al. (2020)
Le Mourre de la Barque	LMB_E10_14CD_C6	Néolithique Moyen MB	14CD	Cereal	*Triticum aestivum/durum/turgidum*	ETH-88883	4934	25	−3770 to 3647	−23.3	22.51	Unpublished

### Archaeobotanical analysis

**Figure 2.**
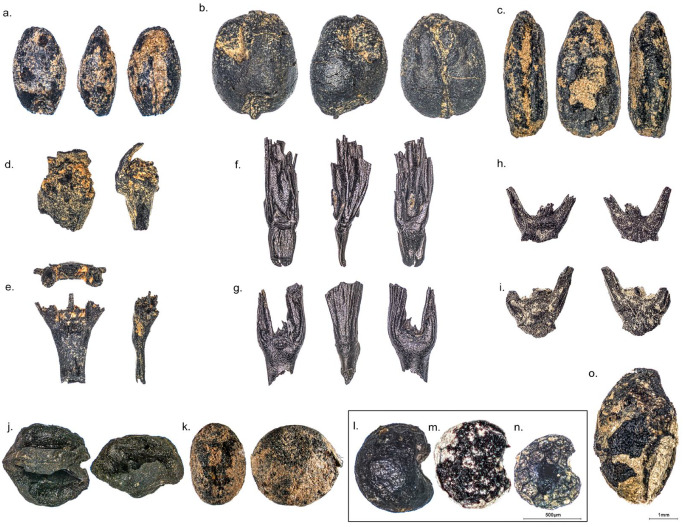
Seed and fruit remains of cultivated crops found in the investigated sites: Cereal grains of (a) *Hordeum vulgare* var. *nudum* (Le Boulou), (b) *Triticum aestivum/durum/turgidum* (Can Sadurní), (c) *Triticum monococcum* (Can Sadurní); chaff remains of (d) *Triticum* cf. *durum/turgidum* (Auvelles), (e) *Hordeum vulgare* var. *nudum* (Auvelles); (f) *Triticum monococcum* (Les Bagnoles), (g) *Triticum* cf. *timopheevii* (Les Bagnoles), (h) *Triticum monococcum* (Cova Freda), (i) *Triticum* cf. *timopheevii* (Cova Freda); Seeds of: (j) *Cicer* cf. *arietinum* (Can Sadurní), (k) *Lens culinaris* (Can Sadurní), (l–n) *Papaver somniferum* (Can Sadurní, Cova Freda and Auvelles) and (o) *Linum usitatissimum* (Can Sadurní) (Photographs by: Raül Soteras).

The focus of this work is on cultivated plants and only the most obvious domesticated cereals, pulses and oil plants will be taken into consideration, despite being aware that some plants, such as goosefoot (*Chenopodium album*) or common vetch (*Vicia sativa*) could have been cultivated at a more or less small scale (Bouby and Léa, 2006; Mueller-Bieniek et al., 2020). Among the cereals, naked barley (*Hordeum vulgare* var. *nudum*), naked wheat (*Triticum aestivum/durum/turgidum*), emmer (*Triticum dicoccum*), einkorn (*Triticum monococcum*) and Timopheevii’s wheat (*Triticum* cf. *timopheevii*) are the most significant. Among naked wheat, hexaploid (*Triticum* cf. *aestivum*) and tetraploid (*Triticum* cf. *durum/turgidum*) might be present, although the latter was clearly dominant. Legumes were mostly represented by lentil (*Lens culinaris*), pea (*Pisum sativum*) and broad bean (*Vicia faba*). Finally, oil plants were identified, including flax (*Linum usitatissimum*) and opium poppy (*Papaver somniferum*) (Figure 2). Waterlogged plant remains were excluded from the analysis, because they were only present at one site, Les Bangoles and they show similar proportions amongst the cereals than the charred assemblage at the site (Jesus et al., 2021). Only two mineralised seeds – of opium poppy (*Papaver somniferum*) – were recovered. Plant impressions on daub, relatively rare in the investigated sites, were also integrated in the dataset.

Crop identification was based on the reference collection available at the IPNA (University of Basel) and a cereal identification atlas (Jacomet et al., 2006). All data were entered into ArboDat (Kreuz and Schäfer, 2014). Cereal grain counts are presented as total counts of remains and not as a minimum number of individuals (grains) (sensu Antolín and Buxó, 2011), since it is not the goal of this paper to establish ratios between grain and chaff (Jones, 1991). Regarding chaff remains, nodes were counted for free-threshing cereals and individual glume bases for glume wheats (Hillman et al., 1996). The category used to amalgamate barley species in graphic representations was *Hordeum distichon/vulgare* (which includes both 2-row and multiple row, as well as naked and hulled barley sorts and identifications of *Hordeum* sp.). Multi-row naked barley dominates in most sites but investigations at Les Bagnoles have showed that 2-row varieties were also present (Jesus et al., 2021). The so-called ‘new’ glume wheat was identified as ‘*Triticum* cf. *timopheevii*’ following the most recent genetic analyses (Czajkowska et al., 2020). All data are available in Supplemental Material 1. Single identifications, such as one find of bitter vetch (*Vicia ervilia*) in Can Sadurní (Antolín and Schäfer, 2020) were not included.

### Radiocarbon dating and stable isotope analysis

A total number of 17 seed and fruit remains (mostly cereal grains but also one wild fruit) from seven sites were radiocarbon dated (Table 1) at the ETH-Zürich Ion Beam Physics Laboratory (LIP). The pretreatment followed the steps of the ABA°60C protocol to remove carbonate and humic acid contamination (Hajdas, 2008). After this chemical cleaning, between 2.2 and 2.4 mg of each sample was selected for AMS radiocarbon dating (if remaining material was available it was used for stable isotope analysis). The dates were calibrated and modelled using OxCal 4.4.4 (Bronk Ramsey, 2021) and the IntCal 2020 Curve (Reimer et al., 2020).

Stable isotope analysis was performed on all dated grains, and additionally on sites with enough charred grains preserved, always following the same pre-treatment mentioned above (Hajdas, 2008). Well-preserved grains were prioritised and, when possible, up to 10 grains per sample were measured individually. Can Sadurní, Pou Nou and Les Bagnoles were the only sites to yield enough grain remains for this type of approach. The majority of measurements were performed on naked barley (*n* = 54) and naked wheat (*n* = 71), followed by glume wheats (*n* = 4). The measurements per phase are relatively similar, around 60 (Table 3). All grains used for radiocarbon dating and stable isotope analyses were photographed on three views and length, width and breadth, as well as weight, were measured before pre-treatment (Supplemental Material 2). The results of all individual measurements are presented in Supplemental Material 3.

Concentration and isotope composition of carbon and nitrogen were determined, at the ETH-Earth Sciences Department laboratory, using a ThermoFisher Flash-EA 1112 coupled with a Conflo IV interface to a ThermoFisher Delta V isotope ratio mass spectrometer (IRMS). Samples were combusted in an oxidation column at 1020°C. The gases were then passed through a reduction column (650°C) yielding N_2_ and CO_2_ which were separated chromatographically and transferred to the IRMS via an open split for on-line isotope measurements. Isotope ratios are reported in the conventional δ-notation with respect to atmospheric N_2_ (AIR) and V-PDB (Vienna Pee Dee Belemnite) standards, respectively. The methods were calibrated with IAEA-N1 (δ^15^N = 0.45), IAEA-N2 (δ^15^N =+20.41) and IAEA N3 (δ^15^N = +4.72) reference materials for nitrogen, and NBS22 (δ^13^C = −30.03) and IAEA CH-6 (δ^13^C = −10.46) for carbon. Reproducibility of the measurements is better than 0.2‰ for both nitrogen and carbon.

The carbon isotope of the grains and the estimated δ^13^C of atmospheric CO_2_ served to calculate the Δ^13^C values (Supplemental Material 3) following Farquhar et al. (1982), Ferrio et al. (2005) and Araus et al. (1997b). Determining the Δ^13^C values of the samples of this study consisted of applying the following formula based on δ^13^C_air_ and plant carbon isotope composition from the CU-INSTAAR/NOAA-CMDL database (http://web.udl.es/usuaris/x3845331/AIRCO2_LOESS.xls).

Histograms, violin and scatter plots were performed with Past4.05 (Ø. Hammer).

## Results

### Archaeobotanical analyses

**Figure 3.**
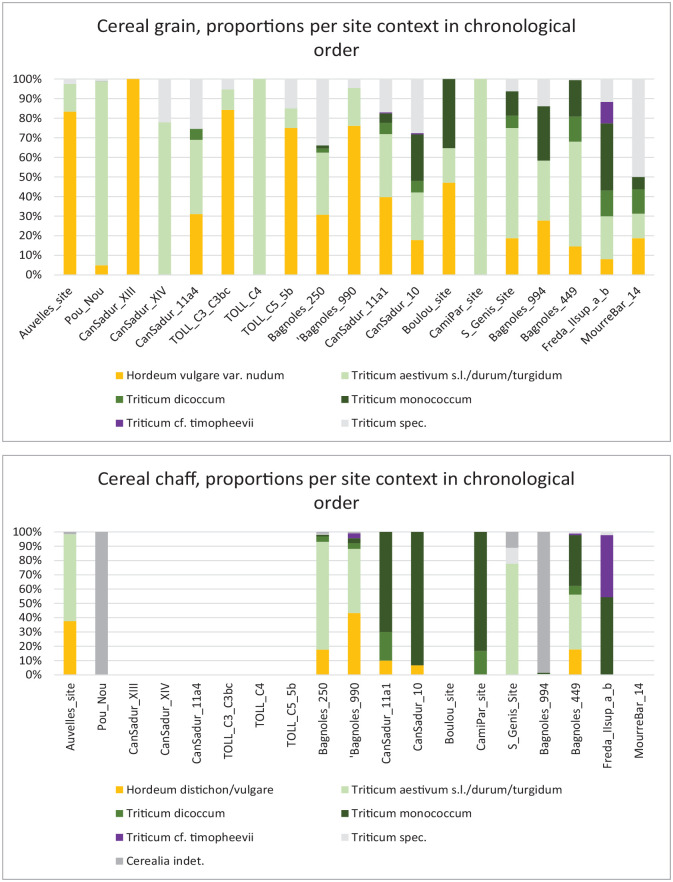
Proportions between the different cereal taxa at a context level and in chronological order. See data in Supplemental Material 1.

The presented results correspond to more than 200 samples, ca. 85 features and more than 4500 L of sediment. Overall, more than 10,000 seed and fruit remains exclusively of cultivated plants were retrieved (Table 2). Data is available in Supplemental Material 1 and partially in Figure 3. The results will be presented in an approximate chronological order, as in Figure 3.

**Table 2.** Summary of the investigated samples per site and phase (Phase 1 = 4500–4000 BC; Phase 2 = 4000–3600 BC) and total of remains of cultivated plants found at the site. In brackets, remains found in a mineralised state.

	Chrono	Sampling –sieving methodology	Number of samples	Number of contexts	Volume (L.)	Number of cultivated plants (charred)
Auvelles	Phase 1	Judgement – wash-over	14	9	>5	1568
Cova del Toll	Phase 1	Unknown – Dry-sieving	15	3		110
Cova Freda	Phase 2	Systematic – wash-over	13	1	123	601
Pou Nou- 2	Phase 1	Systematic – flotation	14	1	>14	2356
Can Sadurní	Phase 1	Systematic – flotation	55	7	3201	2755 (102)
Camí del Paradís	Phase 2	Systematic – water screening	11	10		11
Le Boulou	Phase 2	Systematic – water screening	49	41	335.45	22
La Teuleria	Phase 1	Systematic – water screening	1	1	10	-
La Teuleria	Phase 2	Systematic – water screening	2	2	>10	51
La Teuleria	Phase 2	Systematic – water screening	imprints	1		15
Les Bagnoles	Phase 1	Systematic – wash-over	65	6	657.7	1790
Les Bagnoles	Phase 2	Systematic – wash-over	18	3	225.2	1082
Le Mourre de la Barque	Phase 2	Systematic – wash-over	8	1		23

Most of the data of the site of Auvelles comes from sample UN88. This sample consisted of a small assemblage of grains of naked barley and naked wheat and a relatively large amount of chaff remains from both taxa. Naked barley is slightly dominating in the grain record, while naked wheat (specifically hard wheat) is better documented in the chaff record. No pulses were found, but one seed of opium poppy was recovered. This single find will not be considered further, since it could be a laboratory contamination.

The contents of the pit sampled in Pou Nou 2 were relatively homogeneous. Naked wheat was dominant in the assemblage, with some grains of naked barley and one single find of opium poppy, which we cannot consider further for the reasons stated above.

In Can Sadurní, two layers of burnt dung were analysed: Structure XIII and Structure XIV. Str. XIII only yielded charred grains of naked barley and mineralised awn fragments. Structure XIV only yielded naked wheat grains and one seed of flax. The corresponding layer where these deposits are embedded (11a4) was also rich in naked barley and naked wheat, while some additional finds of einkorn and emmer were also encountered. Among the pulses, pea and fava bean were recovered and scanty finds of flax and opium poppy were also recorded. In the youngest phases (11a1 and 10), even though barley and naked wheat are quantitatively more important, emmer and particularly einkorn and possibly Timopheevii wheat become more visible (only grains of Timopheevii wheat were recovered and hence the identification is insecure). To the suite of legumes and oil plants we observe the main taxa from the older deposits being still present, with the additional documentation of lentil.

The samples from Cova del Toll yielded larger amounts of naked barley and some finds of naked wheat, with one of the highest numbers of pea (*n* = 19) found in the study area. We cannot exclude the presence of opium poppy or flax at the site, since no conventional flotation of sediments was undertaken at the time of the sample recovery and no small finds were recovered.

**Figure 4.**
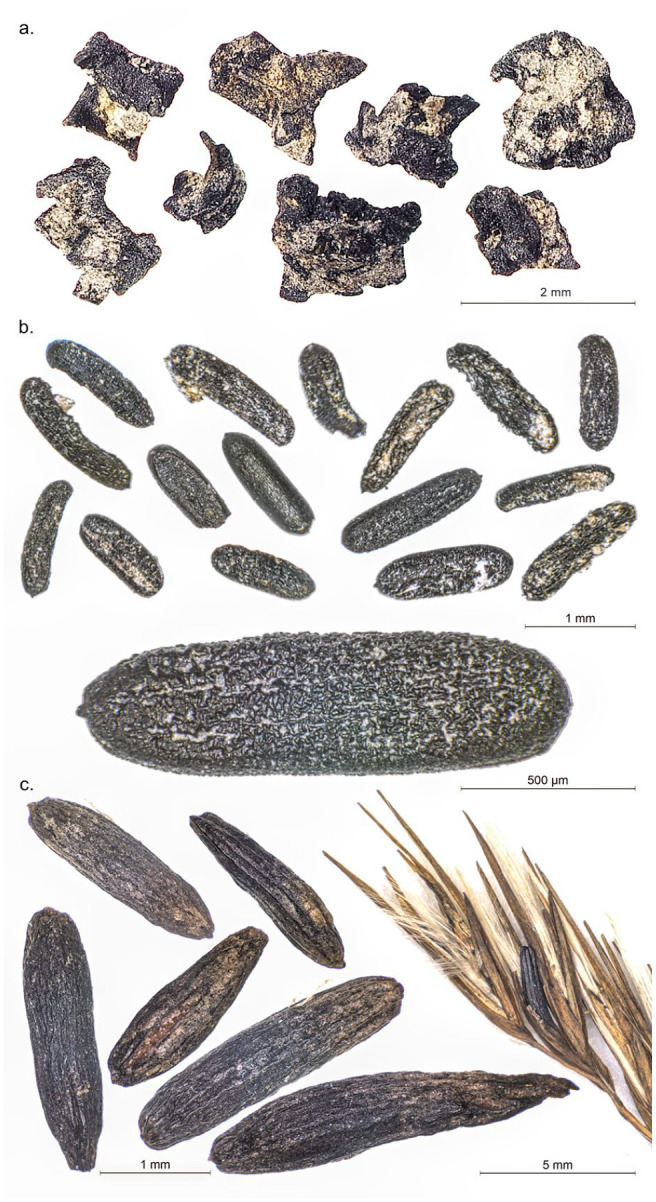
(a) Chaff remains found in structure 449 of Les Bagnoles showing homogeneous poor preservation conditions, (b) Unidentified remains: potential sclerotia of fungi affecting cereal ears and (c) recent sclerotia of ergot (*Claviceps purpurea*) gathered by Örni Akeret (University of Basel). Photographs by: Raül Soteras.

At Les Bagnoles, most of the findings come from the three wells that were excavated, since the preservation conditions are significantly better in waterlogged sediments. Additionally, feature 449 provided a good number of finds. This feature was a pit with numerous burnt bones. The oldest well (250) yielded a lower amount of charred remains, mostly belonging to naked barley and naked wheat. Only a few finds of einkorn were documented. A total of three lentils were found in its deposits. Well 990 dates slightly younger than the previous well, close to 4000 BC. Here significantly more remains were found, still dominated by naked barley and naked wheat, but a larger amount of glume wheats were also recovered in the chaff record (emmer, einkorn and Timopheevi’s wheat). Some charred remains of flax were also found, along with one fava bean. In the third well (Str. 994) less naked wheat and naked barley were present, while glume wheats were overall better represented. Pea was the only legume found. In structure 449 large numbers of badly preserved free-threshing rachis remains were found with some potential remains of sclerotia that we were not able to identify (Figure 4). Glume wheats were also present in the sample, albeit not in higher amounts than naked wheat.

Le Boulou yielded a small number of remains, dominated by naked barley and einkorn, with the presence of naked wheat as well.

The site of Camí del Paradís was poor in finds, but they all belong to wheat, mostly einkorn (identified as chaff), but also emmer and naked wheat.

The site of La Teuleria (S_Genis) allowed the identification of both macroremains and imprints in daub. The results are consistent and indicate that a combination of naked barley, naked wheat, einkorn and emmer was present at the site, being naked wheat slightly better represented than the rest.

Cova Freda yielded a cereal-dominated assemblage with some remains of barley and naked wheat but mostly remains of einkorn, emmer and Timopheevii’s wheat (including both grains and chaff of einkorn and Timopheevii’s wheat). Additionally, some remains of pea, fava bean and 11 seeds of opium poppy were also recovered.

At Le Mourre de la Barque only a few cereal remains were found, with presence of barley, naked wheat, emmer and einkorn and possibly also pea.

### Stable isotope analysis and grain size

**Figure 5.**
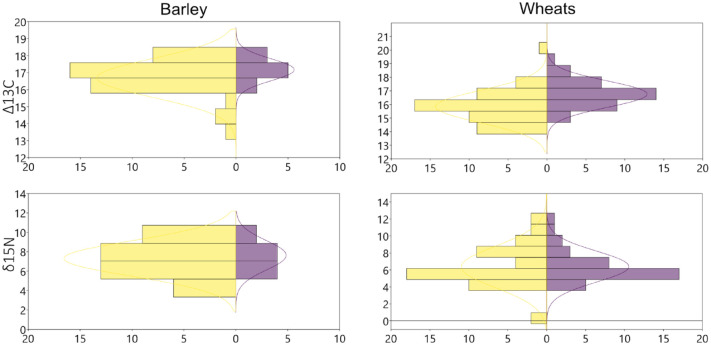
Histograms showing the frequency of carbon and nitrogen isotope values per taxon classified per chronological period: before 4000 BC (yellow) and after (purple). *Note*. Please refer to the online version of the article to view this figure in color.

All results are presented in Supplemental Material 3 and the summarised values per site and phase can be seen on Tables 3 and 4. Carbon stable isotope fractionation between plants and CO_2_, when observed globally, do not show changes before and after 4000 BC in barley but wheats seem to indicate slightly wetter conditions after 4000 BC, with a shift from ca. 16‰ to 17‰. Nitrogen values do not show any changes between both phases in any of the two taxons. Barley tends to slightly higher values (ca. 8‰) than wheats (ca. 6‰) (Figure 5). We observed no correlation between nitrogen isotope values and grain length (Supplemental Material 4) and, likewise, no correlation between carbon and nitrogen isotope values (Supplemental Material 5).

**Table 3.** Summarised results of the stable isotope analysis on charred grain per site and phase.

	Chronology (cal BC)	Barley			Naked wheat			Glume wheat		
		N	Δ^13^C (‰)	δ^15^N (‰)	N	Δ^13^C (‰)	δ^15^N (‰)	N	Δ^13^C (‰)	δ^15^N (‰)
Auvelles	4300–4000	1	18.3	8.6						
Pou Nou	4200–4000	8	16.6 (15.8–17.5)	6.3 (5.2–7.8)	10	14.7 (13.8–15.2)	9.6 (7.7–12.3)			
Can Sadurní	4500–4000	17	16.5 (14.0–18.5)	6.82 (3.3–10.6)	29	16.0 (14.4–18.0)	5.34 (−0.3–9.2)			
Le Boulou	3900–3700	1	167	6.0				1	17.6	5.4
Bagnoles_250	4300–4100	8	16.8 (13.1–18.4)	8.2 (6.7–10.7)	8	16.6 (14.7–20.6)	6.3 (4.5–8.7)			
Bagnoles_990	4100–4000	9	17.2 (16.0–18.3)	8.3 (6.8–9.4)	2	17.5 (17.2–17.7)	6.8 (6.0–7.6)			
Bagnoles_449	3950–3750	10	16.7 (15.7–17.8)	5.8 (4.8–7.6)	17	16.6 (14.9–18.7)	5.7 (4.0–7.3)			
Bagnoles_994	3950–3750				5	17.4 (16.2–19.0)	9.4 (5.4–12.7)	3	17.1 (16.6–17.9)	7.5 (6.0–8.9)

**Table 4.** Summarised results of the grain measurements: average, minimum and maximum values (in mm).

	Barley				Naked wheat				Glume wheat			
	N of grains	Length	Width	Height	N of grains	Length	Width	Height	N of grains	Length	Width	Height
Auvelles	1	4.596	3.359	2.244								
Pou Nou	6	2.76 (2.54–3.09)	1.91 (1.59–2.16)	1.40 (1.25–1.65)	10	3.16 (2.79–3.61)	1.79 (1.57–2.04)	1.69 (1.52–1.90)				
Can Sadurní	9	4.39 (3.27–5.59)	3.14 (2.24–3.88)	2.23 (1.75–2.75)	21	3.82 (2.71–4.70)	2.69 (3.29–1.70)	2.29 (1.45–3.04)	1	6	3.532	3.035
Le Boulou	1	3.223	1.934	1.387					1	3	1.809	2.212
Les Bagnoles_250	5	3.32 (2.62–4.4)	2.3 (1.66–3.2)	1.60 (1.33–2.17)	7	3.06 (2.47–3.23)	2.07 (1.46–2.61)	1.85 (1.53–2.24)				
Les Bagnoles_990	6	3.74 (3.34–4.26)	2.22 (1.83–2.50)	1.67 (1.33–2.17)	2	3.57 (3.43-3.71)	2.25 (2.11–2.38)	2.15 (2.11–2.19)				
Les Bagnoles _449	9	3.92 (2.81–5.32)	2.32 (1.86–2.95)	1.68 (1.25–2.77)	10	4.29 (3.58–4.95)	2.74 (2.47–3.19)	2.39 (2.06–2.83)	17	4.07 (3.48–4.53)	1,76 (1.03–2.45)	1.79 (1.60–2.09)
Les Bagnoles_994					3	3.36 (3.04–3.77)	2.00 (1.50–2.31)	1.87 (1.67–2.01)	3	3.95 (3.58–4.52)	1,93 (1.78–2.16)	1.84 (1.36–2.21)

Only one isotope measurement was performed for the site of Auvelles, on a barley grain that was also dated. With a result of the Δ^13^C of 18.3 ‰, it is amongst the highest values in the Catalan region, yet of little representativeness, since it is just one measurement. The δ^15^N obtained from the same grain was 8.6 ‰, a particularly high value in combination with a high Δ^13^C value. This grain was comparatively also large in size.

The results obtained from Pou Nou 2 concern both naked barley (*n* = 8) and naked wheat (*n* = 10) grains. Δ^13^C values for barley were low (16.6‰ in average) but the lowest values were observed in naked wheat (14.7 ‰ in average). δ^15^N were higher for naked wheat (9.6 ‰ in average) than for naked barley (6.3 ‰ in average). Both barley and naked wheat grains where among the smallest in size of all measured sites.

Can Sadurní presented similar values of Δ^13^C and δ^15^N to those of Pou Nou 2 concerning barley, but the naked wheat yielded somewhat higher Δ^13^C (16.0 ‰ in average) and lower δ^15^N (5.4‰ in average). In contrast to this, the barley grains at Can Sadurní were significantly longer (about 60%) and the naked wheat grains also slightly larger (about 20%).

At Le Boulou only one barley grain was analysed, with values similar to those obtained in Pou Nou and Can Sadurní and a size similar to the grains of Pou Nou 2. One grain of einkorn measured yielded 17.8 ‰ for Δ^13^C and 5.4 ‰ for δ^15^N and was comparatively very small in size.

The data obtained for Les Bagnoles covers the period before and after 4000 BC and has been divided per feature. Barley grains yielded similar Δ^13^C values along the site duration (with average values around 17‰), but showed decreasing δ^15^N values (from 8.3‰ to 5.9‰ in average) in the youngest phase after 4000 BC. Grain size shows different trends, since the average length of barley grains increases from 3.32 to 3.92 mm. Wheats show similar carbon isotope ratios to barley at the site, which is not commonly the case, since wheat usually ripens later and hence suffers more from the summer droughts and tends to have lower carbon isotope discrimination. Nitrogen isotope ratios show the opposite trend than barley: they increase in the youngest phase, but only in the samples from the well 994, reaching 9.4 ‰ in average for naked wheat and 7.5 ‰ for glume wheats. This is not coincidental with grain size, since grains from Feature 449 are larger than the rest despite having the lowest carbon and nitrogen values.

**Figure 6.**
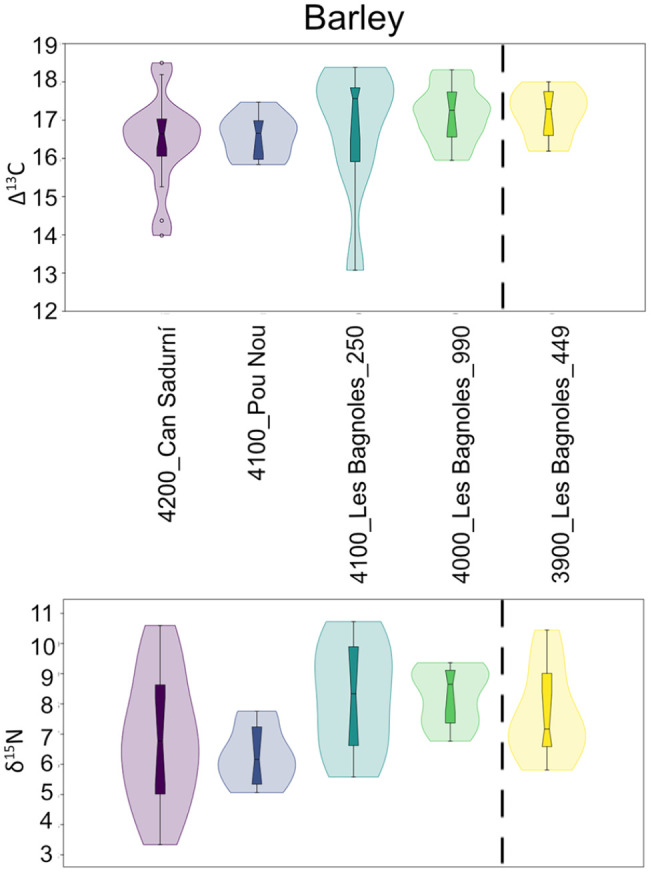
Stable isotope analysis of barley grains in different sites of the study area (top: carbon; bottom: nitrogen). Sites ordered in chronological sense, from older to younger, dashed line indicating 4000 BC.

**Figure 7.**
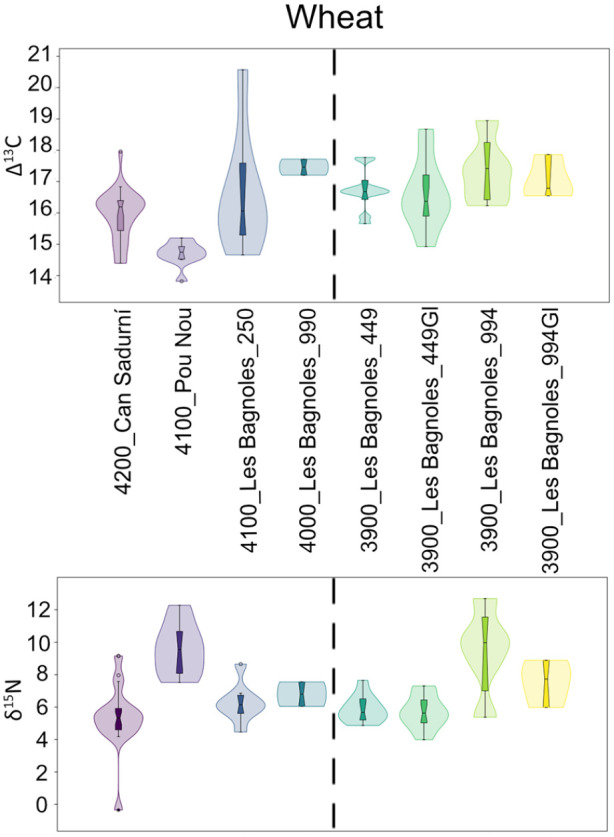
Stable isotope analysis of wheat grains in sites of the study area. Empty boxplots indicate glume wheats (Gl). Sites ordered in chronological sense, from older to younger, dashed line indicating 4000 BC.

**Figure 8.**
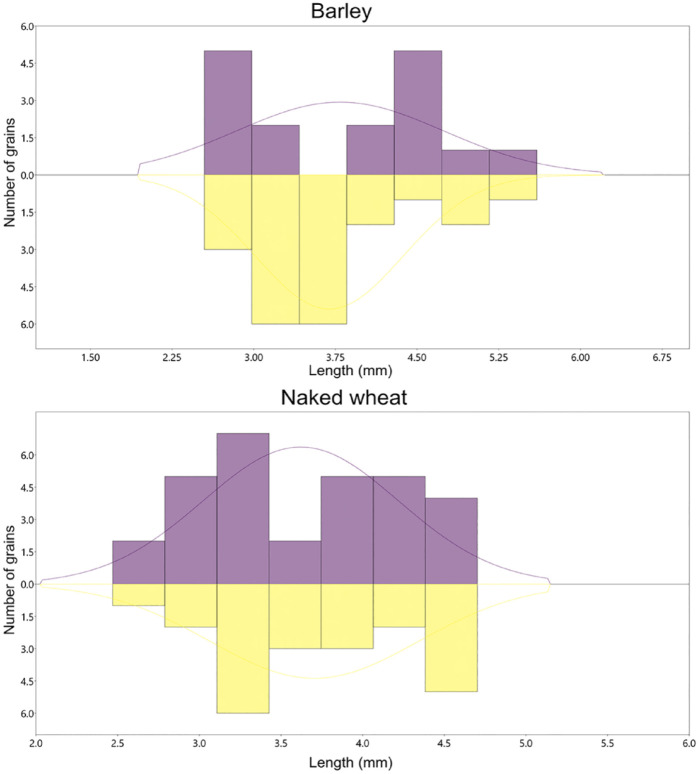
Histograms presenting the length of the grains of barley (top) and wheat (bottom) for the southern sites (purple) and the northern sites (yellow). *Note*. Please refer to the online version of the article to view this figure in color.

When comparing the different sites, we observed clear differences in carbon isotope ratios of barley between the southern and the northern sites, but also higher nitrogen isotope ratios in Les Bagnoles in comparison to Can Sadurní and Pou Nou (Figure 6). Barley grain size in northern sites seems smaller than in southern sites in general terms, yet there seems to be two different size groups in the south (Figure 8). When comparing wheat carbon isotope ratios some oscillations can be observed but no clear trend other than the regional differences between northern and southern sites (with extremely low values in Pou Nou, for instance) (Figure 7). Nitrogen does generate a different pattern, with very high values in well 994 in Les Bagnoles in comparison to other sites (excluding Pou Nou 2, where very high values have also been encountered). These values particularly concern naked wheat, but also glume wheats. Grain size of wheats does not show any geographical correlation.

## Discussion

### Methodological discussion

The dataset available combines samples from archaeological features of different kind, from cave deposits related to animal penning to wells or impressions on daub, and even potential burial contexts such as in Pou Nou or Les Bagnoles (well 250). There could therefore be a risk that the results were driven by the type of context and that the samples were not representative of the economic dynamics at a local level. Quite the opposite, the patterns obtained when comparing the data chronologically (Figure 3) are consistent. Glume wheats are almost absent during the period between 4500 and 4000 BC and only become relevant again towards the change of millennium with few exceptions within the investigated sites. It is worth reminding that the dataset includes few sites in the north analysed for the earlier period and few sites in the south for the later period, and hence that geographical differences may be driving our interpretations. On the one hand, there are actually few sites archaeobotanically investigated in Catalonia that date to the period between 4000 and 3500 BC. The data here presented from Cova Freda is hence a novelty. On the other hand, some data from Southern France on the period 4500–4000 BC from sites such as Encombres or ZAC-Saint Antoine II (Martin et al., 2016) confirm the trend shown by Les Bagnoles, while other sites such as Le Champ du Poste seem to have more glume wheats already before 4000 BC. Regarding the latter, radiocarbon dating the grains directly would be desirable before the site is integrated into the current debate. In conclusion, despite the gaps in the dataset, the new data presented in this paper opens a new scenario for the reconstruction of agricultural practices around 4000 BC in the northwestern Mediterranean arch.

The problem of sample availability per phase is a bit more noticeable due to the lack of isotope data for Catalonia in the fourth millennium BC, and due to few data for France in the fifth millennium BC. It is clear that more data are needed to understand agricultural practices at a site scale. For this purpose, isotope measurements of unmanured and manured crops from the different analysed climatic regions would help us better understand prehistoric values, yet these reference datasets are still to be created. Additionally, aridity seems to have affected the nitrogen isotope values at some sites such as wheat in Pou Nou 2, where wheat grains yielded high values (over 9‰) along with very low carbon isotope values (ca. 14 ‰) and tiny grains, indicating poor growing conditions. Despite these limitations, our research question can still be targeted with the available data because we can observe if (1) there is a trend in the carbon stable isotope values that can be interpreted as due to changes in precipitation or (2) if nitrogen stable isotope values decrease, suggesting soil fertility decrease and impoverishment.

The record of pulses and oil plants is still limited and it becomes difficult to trace patterns. Even single finds of opium poppy seeds must be discarded, given the possibility that they stay stuck in the mesh of the sieve and that unwanted inter-site contaminations take place in the lab. Despite these limitations, pea and opium poppy seem to be present at several sites and we can probably consider them common crops at most sites. Flax was present in the wells of Les Bagnoles, probably because of the excellent preservation conditions in waterlogged sediments (Jesus et al., 2021), and in Can Sadurní, presumably because of the combination of a very extensive sampling strategy (>3000 L of sediment sieved) and good charring conditions in the dung-rich accumulations. One could also propose that it was more commonly cultivated than the data could suggest.

### Agriculture in the NW Mediterranean around 4000 BC

Until recently, agricultural practices in the study area were known at a very coarse level. In the Catalan region, the dominance of free-threshing cereals was clear but it was believed to last until well advanced the fourth millennium BC (Antolín et al., 2015). This is probably due to the lack of radiocarbon dating of Middle Neolithic dwelling contexts (in opposition to funerary contexts, which are very well dated). Further dating of previously investigated sites should be undertaken in order to integrate all available data from this period within a precise and reliable chronology. In some parts of Southern France, it was also believed that einkorn only becomes important after 3500 BC (Bouby et al., 2020b), partly due to the lack of direct dating of most of the archaeobotanical assemblages.

The data here presented indicate that most sites dated between 4500 and 4000 BC in the NW Mediterranean regions based their agriculture in the cultivation of free-threshing cereals, namely naked wheat and naked barley, along with pea, opium poppy and possibly also flax. Free-threshing cereals become widespread in the area around 5100 BC, a characteristic that differentiates farming practices in this area from those in the Balkans and central Europe, where glume wheats are dominant (Antolín et al., 2021, 2022; de Vareilles et al., 2020; Kreuz and Marinova, 2017). It is surprising that some sites such as Auvelles or Les Bagnoles yielded high amounts of chaff remains, even though we are dealing with free-threshing cereals. This is probably indicating that the fields were located very close to the settlements, if not within the settlements and that threshing was performed on site.

Stable isotope values presented here indicate that barley usually has higher δ^15^N values than wheat, which could mean that it received more manure and/or grew on better soils. Some preliminary analyses, such as the one at the site of Auvelles would support this hypothesis, since the barley grain analysed showed high carbon, nitrogen isotope values and bigger size. However, higher nitrogen values did not significantly correlate with larger grains (Supplementary Material 4). Other factors affecting nitrogen isotope values such as aridity should be considered (Styring et al., 2017a). We cannot be sure why barley would have been prioritised over wheat. It is possible that it was used for the production of fermented beverages like beer, and perhaps for this reason it could have been grown on richer soils, as it has been observed elsewhere (Styring et al., 2017b). Beer production has been proposed for the site of Can Sadurní (Blasco et al., 2008), but there is not conclusive evidence for it.

Pou Nou 2 is a site that would be interesting to explore further in the future. The small amount of grain found shows very poor growing conditions (low nitrogen isotope values and small size of the grains). This grain comes from the richest sample in acorn remains of the Neolithic in the Northeast of the Iberian Peninsula, estimated in 40–70 kg (Antolín et al., 2018b). It might be indicative of a case of complementary gathering of wild resources in years of crop failure.

After 4000 BC einkorn, emmer and Timopheevi’s wheat became widespread in both areas. Timopheevi’s wheat has only recently been identified in Southern France (Jesus et al., 2021). A revision of material investigated in the previous decades might become necessary for its reliable identification, as this cereal has only been recognised since a relatively short time (Jones et al., 2000; Kohler-Schneider, 2003). Naked wheat is still present in many sites, sometimes in relatively high amounts (Figure 3), but glume wheats achieve significant proportions that were not observed previously. Some of these crops could have arrived via exchange with other regions, such as North-eastern Italy, but the available isotope values obtained at Les Bagnoles indicate similar growing conditions to those of naked wheat. The δ^15^N values obtained at this site for naked wheat and glume wheats indicate new priorities in the farming system (with wheats replacing barley in importance), and possibly an attempt to improve the harvest of naked wheat by intentionally increasing the nutrients available to the crop. According to the archaeobotanical data available naked wheat was not the most successful crop despite having received (presumably) the best soils, since its presence in the record reduces significantly in comparison to earlier phases.

### Analysing an agricultural change

The crop diversity of the late fifth millennium cal. BC, with naked wheat and naked barley, pulses and opium poppy was relatively reduced and presumably poorly resilient in front of certain factors.

Among the reasons postulated to explain the shift from free-threshing to non-free-threshing cereals, authors have discussed soil impoverishment, climate change, new networks and the spread of pests. We will discuss each factor separately.

Soil impoverishment is a frequent argument in archaeology to explain socioeconomic changes. Nevertheless, clear evidence is not always found. We would expect decreasing δ^15^N values as an indication for a progressive loss of nutrient of the soils. On the contrary, we observe either the maintenance or the increase of δ^15^N values (Figures 4 and 5). Even though the sites being compared present differences that do not allow fine-scaled comparisons, our results indicate no evidence of soil impoverishment in the region and we concur with other researchers in saying that soil fertility was probably not the most important limiting factor in early agriculture (Halstead, 1999).

Climate change has of course been another important explaining factor for many socioeconomic processes. Tetraploid naked wheat, dominant in the Western Mediterranean since the end of the sixth millennium cal BC (Antolín, 2016), is well-adapted to the Mediterranean dry summers. In this sense, a change towards the growing of glume wheats might be related to wetter or colder climatic conditions. Palaeoclimatic data is still rarely available at a local level and is hence difficult to adapt to the human temporal and geographical scales. A reconstruction of temperatures for the past 17,000 years was recently published for the southern Massif Central in southern France (d’Oliveira et al., 2023). This study managed to detect a cooling period after the so-called Holocene Thermal Maximum, from ca. 4600 to 3000 cal. BC, that is also attested in other records of the Swiss Alps, Northern Italy and Spain (d’Oliveira et al., 2023; Jalali et al., 2016). Tetraploid naked wheat would indeed be sensitive to low winter temperatures and glume wheats would have been more resilient in front of such a climatic change. It is nevertheless currently unclear if this cooling period could have affected winter temperatures and there is currently no chronological correlation with the sudden change in agricultural practices around 4000 BC observed in the archaeobotanical record. In comparison to temperature reconstructions, available proxies for precipitation for the time period of interest are rare. For instance, local records of high importance for the seventh and sixth millennia cal. BC lack deposits for the time period of our interest (i.e. Berger et al., 2016). We can nevertheless also rely on nearby records, since previous research in the area show that local trends observed in river catchment areas coincide with broader climatic dynamics in the central and western Mediterranean (Berger et al., 2016). A high-resolution palaeoclimatic record from the Adriatic Sea detects a stability phase of higher temperature and slightly higher rainfall during the period between ca. 4500 and 3500 BC (Combourieu-Nebout et al., 2013). Similarly, favourable conditions for the same time period are interpreted from several archaeobotanical indicators from sites in Southern France (Delhon et al., 2009). A progressive decrease in summer precipitation is nevertheless plausible, leading to a decrease of deciduous forests after 2500 BC (Combourieu-Nebout et al., 2013), but the onset of this process is unclear, and some authors postulate maximum rainfall levels were reached between 4000 and 1000 BC (i.e. Magny et al., 2013). Actually, Jalut and others define this time period as the transition phase between the early and the Late-Holocene (Jalut et al., 2009). The most significant shift observed to date in the area seems to be the stabilisation of the sea level detected in the river mouth of the Hérault and other rivers, and the onset of deltaic developments (i.e. Devillers et al., 2019), but this is probably not only related to climatic factors. While we can assume that these changes could have impacted crop growth on the long run, it is hardly possible to correlate them with the rapid change observed in the archaeobotanical record. For this reason, we resorted to stable isotope analyses of cereal grains as a harvest-scale resolution proxy of water availability (Fiorentino and Caracuta, 2015) for the samples that we investigate archaeobotanically – and hence suffering from the same problems of representativeness. The carbon values obtained concur with the palaeoclimatic data presented above and do not support a significant change in spring rainfall in the studied area. Instead, they seem to reflect climatic differences between sites, similar to the existing differences nowadays in the study area (they are mostly between 300–500 mm of yearly rainfall). Although altitude can affect carbon isotope values (Körner et al., 1988) the altitude difference between 50 and 450 m asl is too small to induce carbon stable isotope variations. In short, the fact that different sites with different rainfall regimes seem to change crops within a short period of time suggests that the climate change was not the decisive factor determining the speed of this change.

Since the combination of emmer, einkorn and Timophevi’s wheat seem to spread in a sort of a ‘package’, it is a logical thought to consider that they arrived via exchange with groups of people that cultivated this suite of crops. At the time, the closest areas where these crops were grown are located in North-eastern Italy (Reed and Rottoli, 2014; Rottoli, 2014; Rottoli and Castiglioni, 2009), and the possibility of long-distance contacts between these areas has already been proposed in previous publications (Antolín et al., in press; Jesus et al., 2021; Steiner et al., in press). At the site of Isolino Virginia (Lake Varese, North-western Italy), we observe a similar shift. While the agricultural practices at the site are based on naked barley, naked wheat, pea, poppy and flax during the fifth millennium BC, a clear shift towards glume wheats is observed after 4000 BC with almost a complete abandonment of naked wheat (Steiner et al., in press).

**Figure 9.**
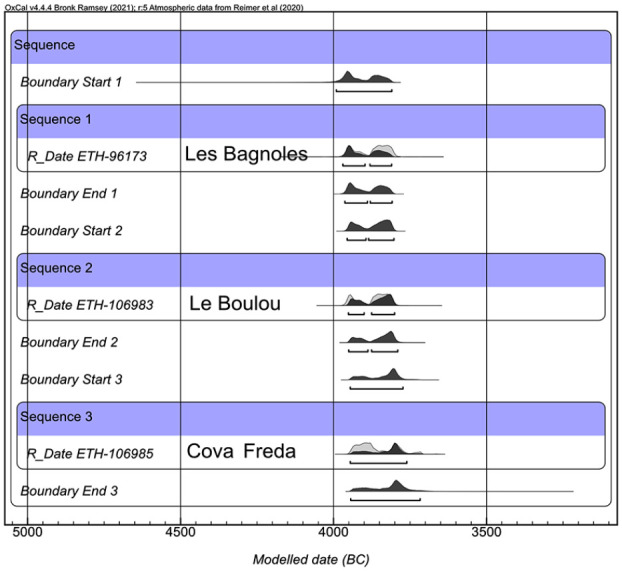
Sequential model of 3 radiocarbon dates on glume wheat grains performed with OxCal v4.4.4.4.

One way to pursue this hypothesis would be to date all evidence of glume wheats from sites from this time period. Until this information is available, it is not possible to properly test whether the available data fits with a westward spread of glume wheats, but we can make a first assessment. The available dates, though scarce, do confirm this trend (Figure 9) and a sequential model performed with OxCal indicates a good level of agreement (Supplementary Material 6), but a lot more dates of einkorn and Timopheevi’s wheat from these and other sites would be desirable for resolving this question or at least reach a better understanding of the complex process that could be behind this economic change. Other ways of approaching this question include ancient DNA analyses of cereal remains from well-preserved sites in order to test whether the cultivars grown in different sites could be related or not, a work that is currently on-going (F. Follmann, PhD at FU Berlin).

Most recently, pests were proposed as one of the key factors to consider as potential drivers of agricultural decision-making processes (Häberle et al., 2022). Despite being one of the major risks for farmers, pests have only rarely been investigated in the archaeology of continental Europe and particularly in the Western Mediterranean. Recent investigations at the site of Les Bagnoles, as well as at the site of Isolino Virginia (Antolín et al., in press) indicate interesting patterns of the appearance of pests before and after 4000 BC. Evidence of pests declines at both sites after the adoption of glume wheats. If pests had really spread across multiple sites, as the finds of Les Bagnoles and Isolino suggest, the reduced set of crops available (naked wheat and naked barley) would pose many challenges, since they are particularly easy for pests to attack (not only insects, but also birds). In such a situation, the adoption of glume wheats might have been perceived as advantageous, despite the additional processing work. Other types of pests have remained less explored in Archaeology, such as fungi. The finds of sclerotia together with badly preserved chaff remains in feature 449 in Les Bagnoles, although not identified, might be indicative of further problems affecting the crop at this site. This must remain a speculation for now.

All in all, given the rapidness of the phenomenon and its wide geographical impact, it is difficult to argue that one single factor can explain the change everywhere. It was probably a more complex process that may have some general patterns and local dynamics. In order to disentangle the local processes, more research is necessary, particularly paying attention to waterlogged deposits, where excellent preservation conditions allow for more complex integrative analyses involving multiple disciplines, that is, archaeoentomology.

## Conclusions

This paper presents a large dataset based on numerous archaeobotanical analyses over the past few years that complement available data for the period between 4500 and 3500 BC in the North-western Mediterranean area. Archaeobotanical data has been complemented with radiocarbon and stable isotope data, which allowed new insights into the question regarding the change of crops around 4000 BC in the area.

The data presented suggested something so-far unknown, which is that the change in crops observed in France at ca. 4000 BC may also be detectable in the NE of the Iberian Peninsula and that therefore this shift in the crop assemblage has a very broad geographical coverage. Another new aspect is that the suite of glume wheats cultivated after 4000 BC includes Timopheevi’s wheat (and not just emmer and einkorn), which had until recently not been detected in Southern France. At a preliminary level, the dating of the assemblages allowed to put forward a westward spread of the glume wheats.

Available evidence supports a rapid spread of glume wheats, which would have been integrated into the local farming systems, yet abandoning naked barley in several sites. The stable isotope data support this shift of priorities at some sites, from naked barley to naked and hulled wheats receiving the best-manured soils, which shows a certain degree of planning. Overall the combined analyses performed in this paper prove useful to answer questions regarding agricultural decision-making in the past.

Given that, parallel to these new agricultural practices, other changes (i.e. in pottery) have been observed, one may argue that contact networks with the East (Northern Italy and the Balkans) might have involved more than just objects and grain. In this sense, it would be interesting to investigate the scale of this process further in the future and integrate other regions where these changes seem to happen some centuries later, such as the Swiss Plateau.

## Supplemental Material

sj-csv-6-hol-10.1177_09596836231211848 – Supplemental material for An archaeobotanical and stable isotope approach to changing agricultural practices in the NW Mediterranean region around 4000 BC Click here for additional data file. Supplemental material, sj-csv-6-hol-10.1177_09596836231211848 for An archaeobotanical and stable isotope approach to changing agricultural practices in the NW Mediterranean region around 4000 BC by Ferran Antolín, Stefanie Jacomet, Raül Soteras, Claudia Gerling, Stefano M Bernasconi, Franziska Follmann, Irka Hajdas, Madalina Jaggi, Ana Jesus, Héctor Martínez-Grau, Francesc Xavier Oms, Brigitte Röder, Bigna L Steiner and Samuel van Willigen in The Holocene

sj-docx-4-hol-10.1177_09596836231211848 – Supplemental material for An archaeobotanical and stable isotope approach to changing agricultural practices in the NW Mediterranean region around 4000 BC Click here for additional data file. Supplemental material, sj-docx-4-hol-10.1177_09596836231211848 for An archaeobotanical and stable isotope approach to changing agricultural practices in the NW Mediterranean region around 4000 BC by Ferran Antolín, Stefanie Jacomet, Raül Soteras, Claudia Gerling, Stefano M Bernasconi, Franziska Follmann, Irka Hajdas, Madalina Jaggi, Ana Jesus, Héctor Martínez-Grau, Francesc Xavier Oms, Brigitte Röder, Bigna L Steiner and Samuel van Willigen in The Holocene

sj-docx-5-hol-10.1177_09596836231211848 – Supplemental material for An archaeobotanical and stable isotope approach to changing agricultural practices in the NW Mediterranean region around 4000 BC Click here for additional data file. Supplemental material, sj-docx-5-hol-10.1177_09596836231211848 for An archaeobotanical and stable isotope approach to changing agricultural practices in the NW Mediterranean region around 4000 BC by Ferran Antolín, Stefanie Jacomet, Raül Soteras, Claudia Gerling, Stefano M Bernasconi, Franziska Follmann, Irka Hajdas, Madalina Jaggi, Ana Jesus, Héctor Martínez-Grau, Francesc Xavier Oms, Brigitte Röder, Bigna L Steiner and Samuel van Willigen in The Holocene

sj-pdf-2-hol-10.1177_09596836231211848 – Supplemental material for An archaeobotanical and stable isotope approach to changing agricultural practices in the NW Mediterranean region around 4000 BC Click here for additional data file. Supplemental material, sj-pdf-2-hol-10.1177_09596836231211848 for An archaeobotanical and stable isotope approach to changing agricultural practices in the NW Mediterranean region around 4000 BC by Ferran Antolín, Stefanie Jacomet, Raül Soteras, Claudia Gerling, Stefano M Bernasconi, Franziska Follmann, Irka Hajdas, Madalina Jaggi, Ana Jesus, Héctor Martínez-Grau, Francesc Xavier Oms, Brigitte Röder, Bigna L Steiner and Samuel van Willigen in The Holocene

sj-xlsx-1-hol-10.1177_09596836231211848 – Supplemental material for An archaeobotanical and stable isotope approach to changing agricultural practices in the NW Mediterranean region around 4000 BC Click here for additional data file. Supplemental material, sj-xlsx-1-hol-10.1177_09596836231211848 for An archaeobotanical and stable isotope approach to changing agricultural practices in the NW Mediterranean region around 4000 BC by Ferran Antolín, Stefanie Jacomet, Raül Soteras, Claudia Gerling, Stefano M Bernasconi, Franziska Follmann, Irka Hajdas, Madalina Jaggi, Ana Jesus, Héctor Martínez-Grau, Francesc Xavier Oms, Brigitte Röder, Bigna L Steiner and Samuel van Willigen in The Holocene

sj-xlsx-3-hol-10.1177_09596836231211848 – Supplemental material for An archaeobotanical and stable isotope approach to changing agricultural practices in the NW Mediterranean region around 4000 BC Click here for additional data file. Supplemental material, sj-xlsx-3-hol-10.1177_09596836231211848 for An archaeobotanical and stable isotope approach to changing agricultural practices in the NW Mediterranean region around 4000 BC by Ferran Antolín, Stefanie Jacomet, Raül Soteras, Claudia Gerling, Stefano M Bernasconi, Franziska Follmann, Irka Hajdas, Madalina Jaggi, Ana Jesus, Héctor Martínez-Grau, Francesc Xavier Oms, Brigitte Röder, Bigna L Steiner and Samuel van Willigen in The Holocene
