# Supplementary material for: An archaeobotanical and stable isotope approach to changing agricultural practices in the NW Mediterranean region around 4000 BC
Source: Holocene. 2023 Dec 4;34(2):239–54. doi: 10.1177/09596836231211848 (PMC10799764; doi:10.1177/09596836231211848)
Supplement: sj-pdf-2-hol-10.1177_09596836231211848 – Supplemental material for An archaeobotanical and stable isotope approach to changing agricultural practices in the NW Mediterranean region around 4000 BC [file sj-pdf-2-hol-10.1177_09596836231211848.pdf]

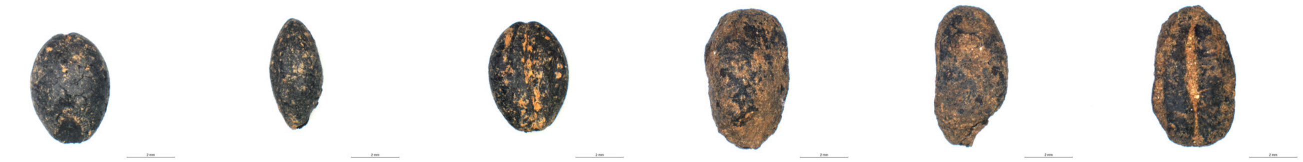

AGRI_C_0090_D AGRI_C_0090_L AGRI_C_0090_V AGRI_C_0093_D AGRI_C_0093_L AGRI_C_0093_V

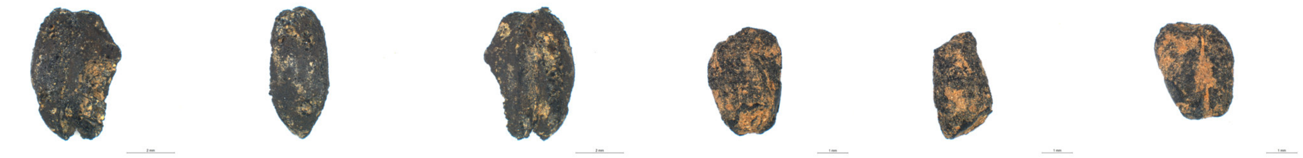

AGRI_C_0095_D AGRI_C_0095_L AGRI_C_0095_V AGRI_C_0099_D AGRI_C_0099_L AGRI_C_0099_V

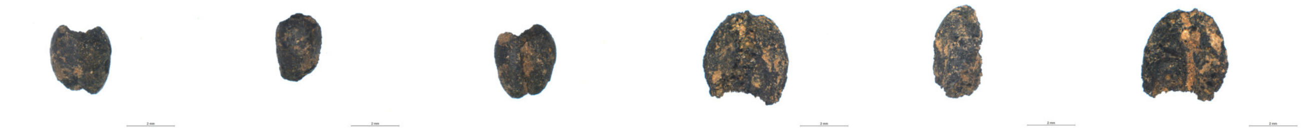

AGRI_C_0172_D AGRI_C_0172_L AGRI_C_0172_V AGRI_C_0173_D AGRI_C_0173_L AGRI_C_0173_V

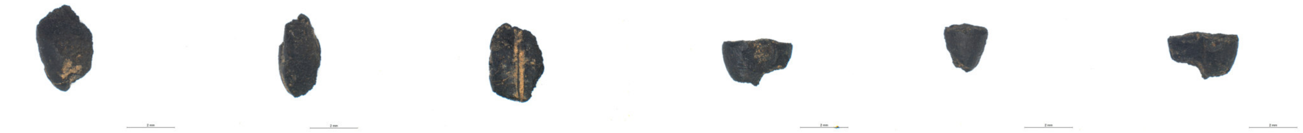

AGRI_C_0174_D AGRI_C_0174_L AGRI_C_0174_V AGRI_C_0175_D AGRI_C_0175_L AGRI_C_0175_V

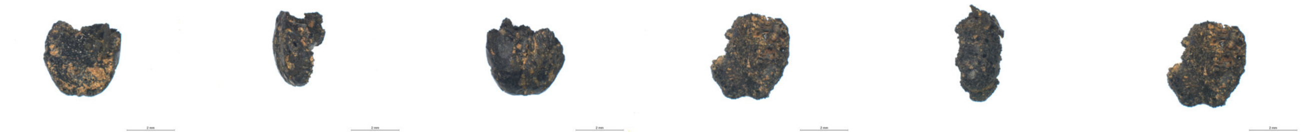

AGRI_C_0177_D AGRI_C_0177_L AGRI_C_0177_V AGRI_C_0178_D AGRI_C_0178_L AGRI_C_0178_V

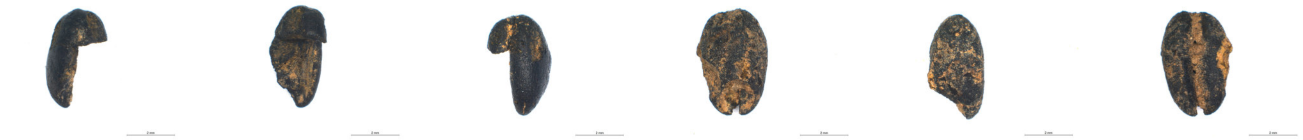

AGRI_C_0179_D AGRI_C_0179_L AGRI_C_0179_V AGRI_C_0180_D AGRI_C_0180_L AGRI_C_0180_V

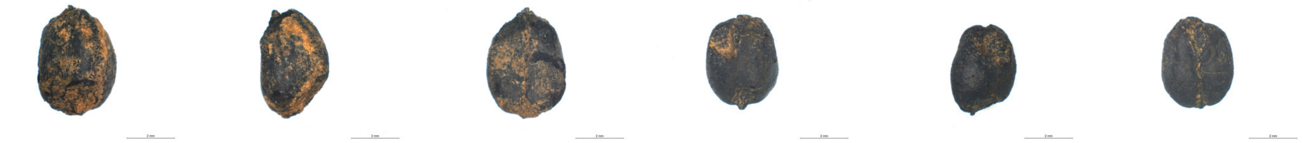

AGRI_C_0181_D AGRI_C_0181_L AGRI_C_0181_V AGRI_C_0182_D AGRI_C_0182_L AGRI_C_0182_V

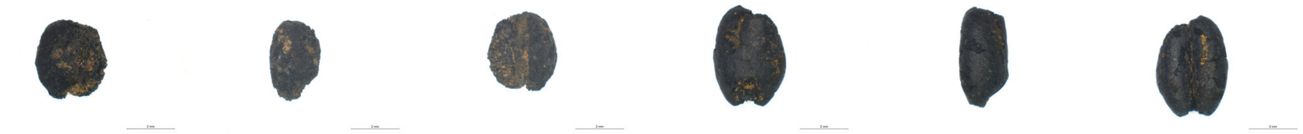

AGRI_C_0183_D AGRI_C_0183_L AGRI_C_0183_V AGRI_C_0184_D AGRI_C_0184_L AGRI_C_0184_V

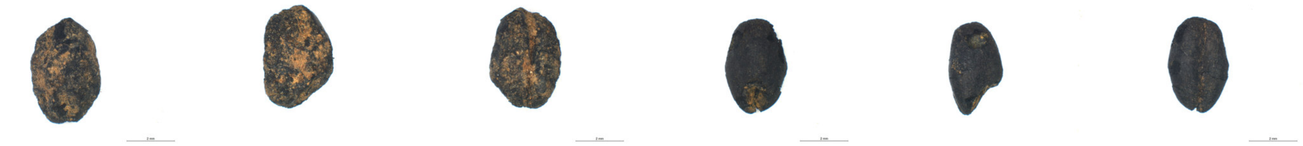

AGRI_C_0185_D AGRI_C_0185_L AGRI_C_0185_V AGRI_C_0186_D AGRI_C_0186_L AGRI_C_0186_V

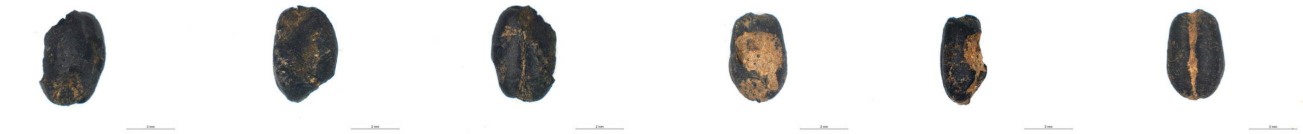

AGRI_C_0187_D AGRI_C_0187_L AGRI_C_0187_V AGRI_C_0188_D AGRI_C_0188_L AGRI_C_0188_V

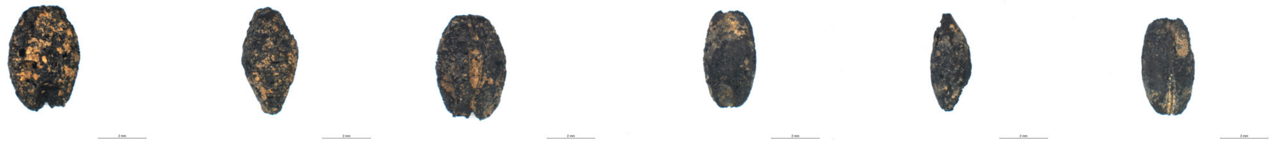

AGRI_C_0189_D AGRI_C_0189_L AGRI_C_0189_V AGRI_C_0190_D AGRI_C_0190_L AGRI_C_0190_V

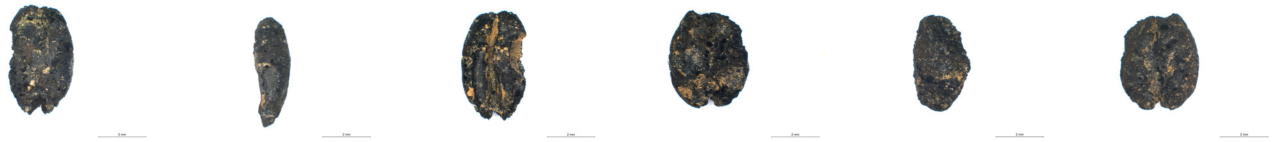

AGRI_C_0191_D AGRI_C_0191_L AGRI_C_0191_V.tif AGRI_C_0192_D AGRI_C_0192_L AGRI_C_0192_V

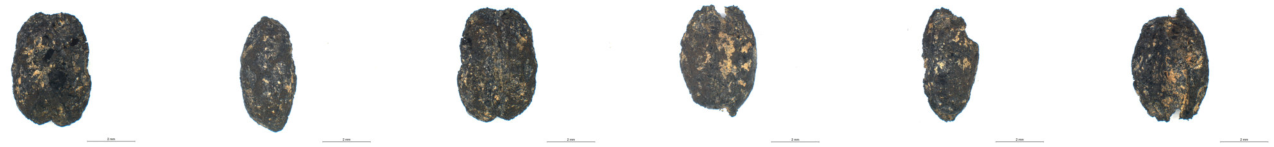

AGRI_C_0193_D AGRI_C_0193_L AGRI_C_0193_V AGRI_C_0194_D AGRI_C_0194_L AGRI_C_0194_V

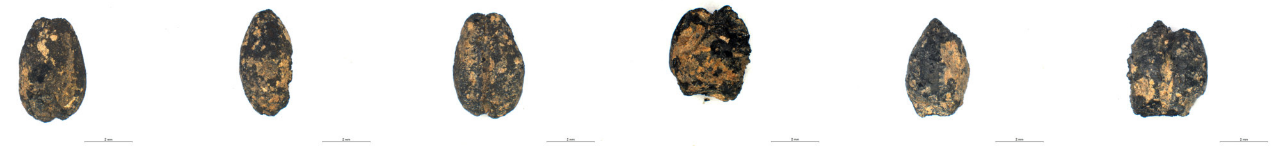

AGRI_C_0195_D AGRI_C_0195_L AGRI_C_0195_V AGRI_C_0196_D AGRI_C_0196_L AGRI_C_0196_V

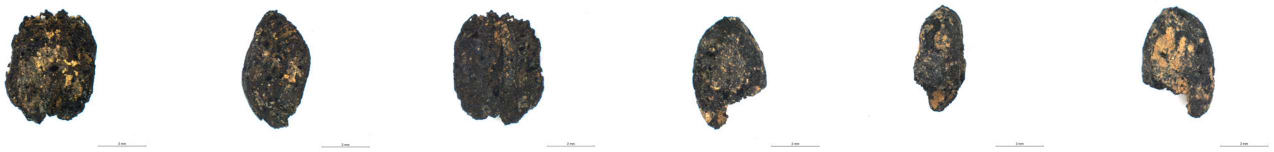

AGRI_C_0197_D AGRI_C_0197_L AGRI_C_0197_V AGRI_C_0198_D AGRI_C_0198_L AGRI_C_0198_V

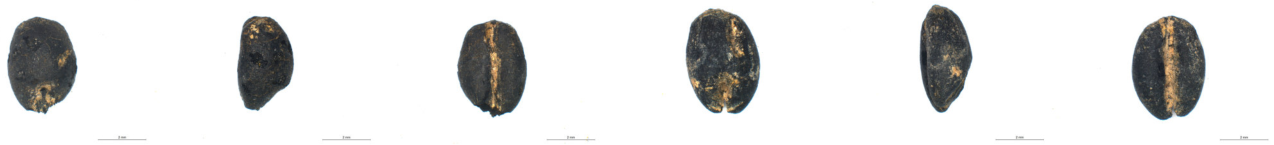

AGRI_C_0199_D AGRI_C_0199_L AGRI_C_0199_V AGRI_C_0200_D AGRI_C_0200_L AGRI_C_0200_V

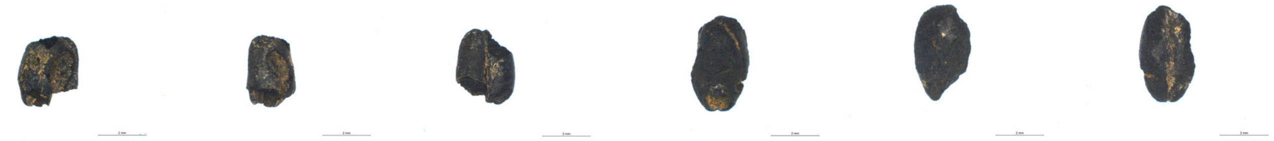

AGRI_C_0201_D AGRI_C_0201_L AGRI_C_0201_V AGRI_C_0202_D AGRI_C_0202_L AGRI_C_0202_V

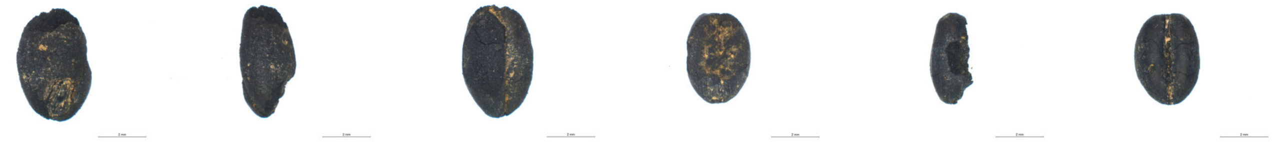

AGRI_C_0203_D AGRI_C_0203_L AGRI_C_0203_V AGRI_C_0204_D AGRI_C_0204_L AGRI_C_0204_V

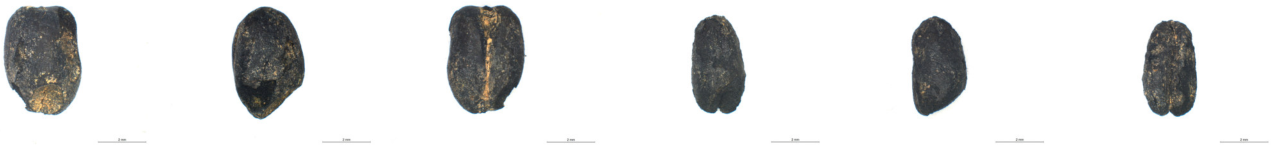

AGRI_C_0205_D AGRI_C_0205_L AGRI_C_0205_V AGRI_C_0206_D AGRI_C_0206_L AGRI_C_0206_V

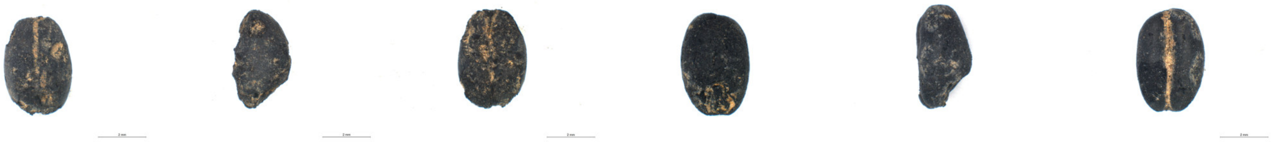

AGRI_C_0207_D AGRI_C_0207_L AGRI_C_0207_V AGRI_C_0208_D AGRI_C_0208_L AGRI_C_0208_V

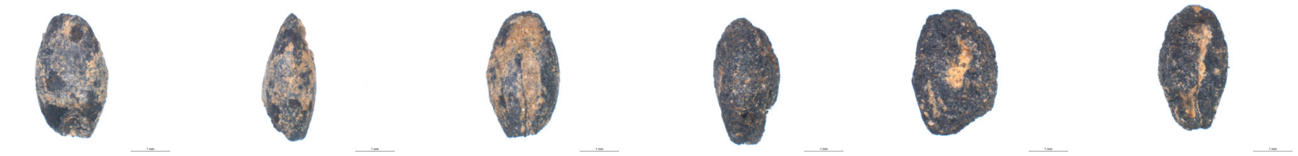

AGRI_C_0372_D AGRI_C_0372_L AGRI_C_0372_V AGRI_C_0373_D AGRI_C_0373_L AGRI_C_0373_V

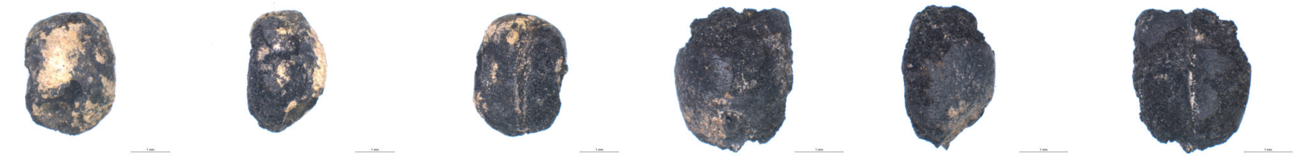

AGRI_C_0512_D AGRI_C_0512_L AGRI_C_0512_V AGRI_C_0513_D AGRI_C_0513_L AGRI_C_0513_V

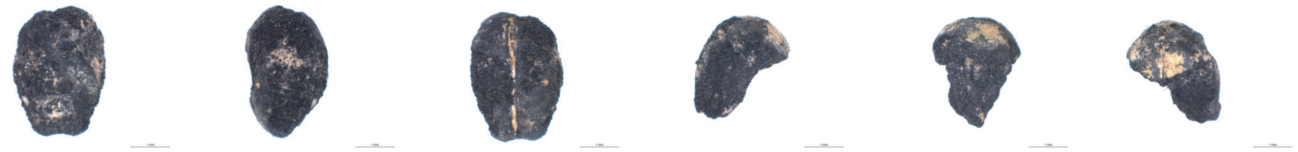

AGRI_C_0514_D AGRI_C_0514_L AGRI_C_0514_V AGRI_C_0515_D AGRI_C_0515_L AGRI_C_0515_V

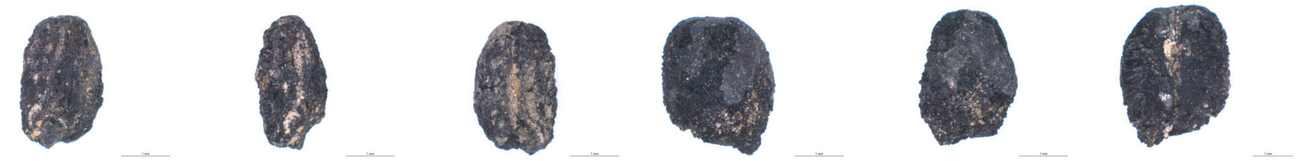

AGRI_C_0516_D AGRI_C_0516_L AGRI_C_0516_V AGRI_C_0517_D AGRI_C_0517_L AGRI_C_0517_V

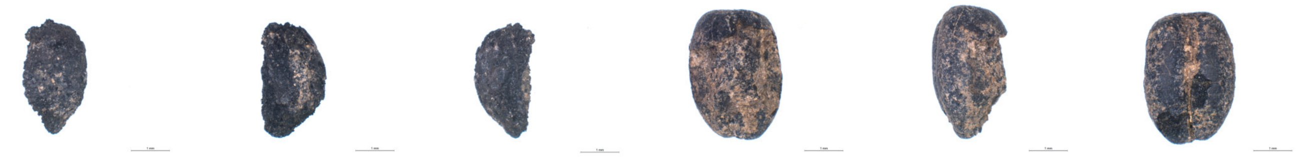

AGRI_C_0518_D AGRI_C_0518_L AGRI_C_0518_V AGRI_C_0519_D AGRI_C_0519_L AGRI_C_0519_V

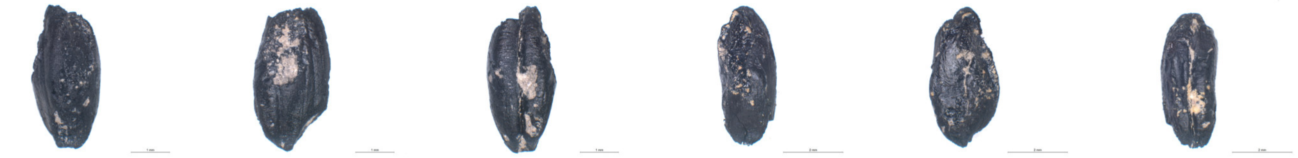

AGRI_C_0534_D AGRI_C_0534_L AGRI_C_0534_V AGRI_C_0535_D AGRI_C_0535_L AGRI_C_0535_V

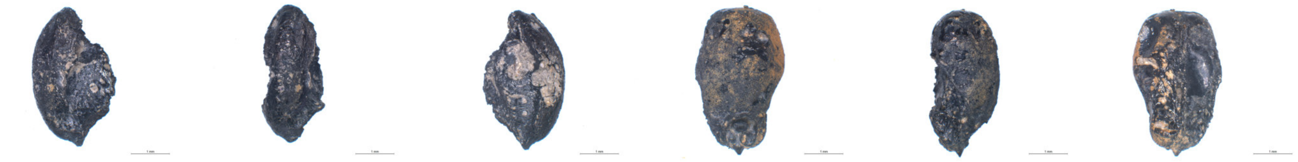

AGRI_C_0536_D AGRI_C_0536_L AGRI_C_0536_V AGRI_C_0537_D AGRI_C_0537_L AGRI_C_0537_V

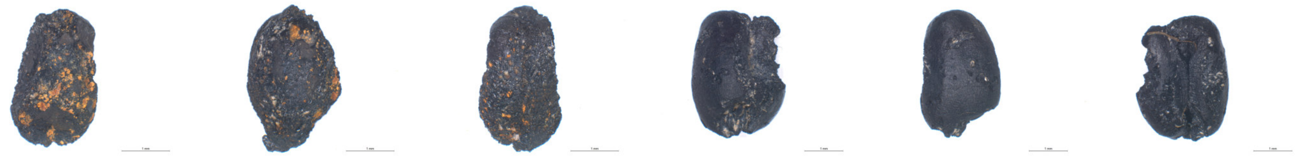

AGRI_C_0538_D AGRI_C_0538_L AGRI_C_0538_V AGRI_C_0539_D AGRI_C_0539_L AGRI_C_0539_V

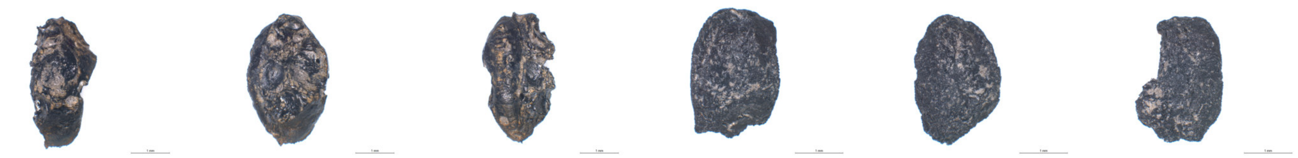

AGRI_C_0540_D AGRI_C_0540_L AGRI_C_0540_V AGRI_C_0541_D AGRI_C_0541_L AGRI_C_0541_V

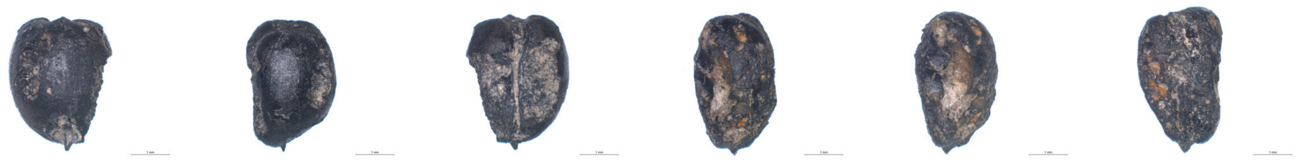

AGRI_C_0542_D.tif AGRI_C_0542_L.tif AGRI_C_0542_V AGRI_C_0543_D AGRI_C_0543_L AGRI_C_0543_V

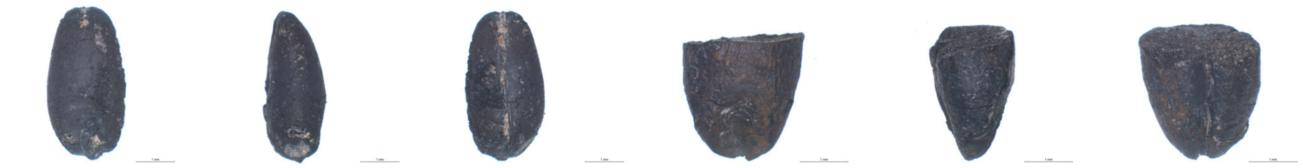

AGRI_C_0544_D AGRI_C_0544_L AGRI_C_0544_V AGRI_C_0545_D AGRI_C_0545_L AGRI_C_0545_V

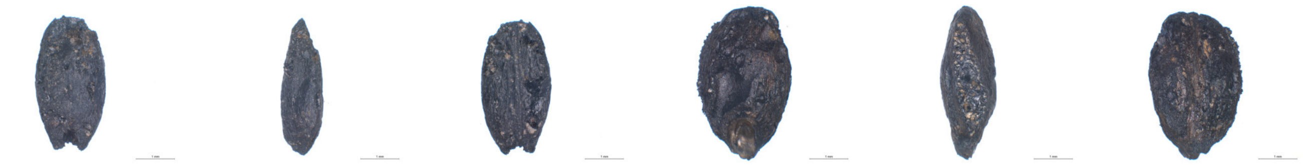

AGRI_C_0546_D AGRI_C_0546_L AGRI_C_0546_V AGRI_C_0547_D AGRI_C_0547_L AGRI_C_0547_V

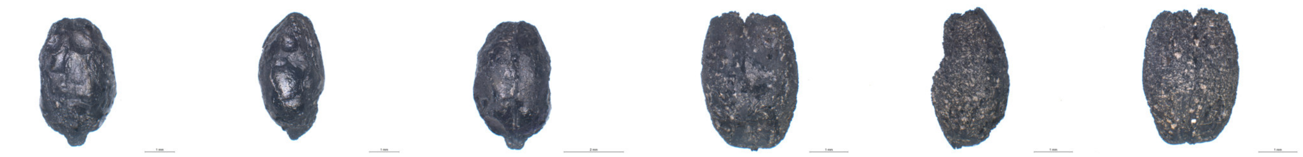

AGRI_C_0548_D AGRI_C_0548_L AGRI_C_0548_V AGRI_C_0549_D AGRI_C_0549_L AGRI_C_0549_V

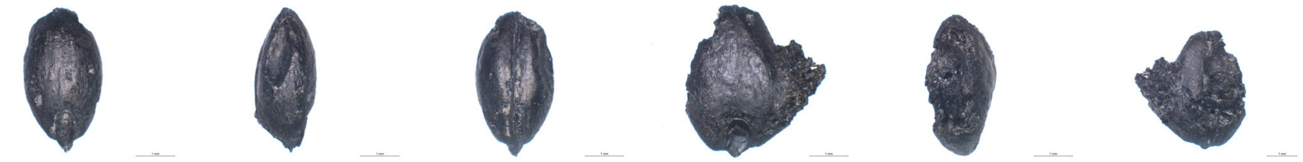

AGRI_C_0550_D AGRI_C_0550_L AGRI_C_0550_V AGRI_C_0551_D AGRI_C_0551_L AGRI_C_0551_V

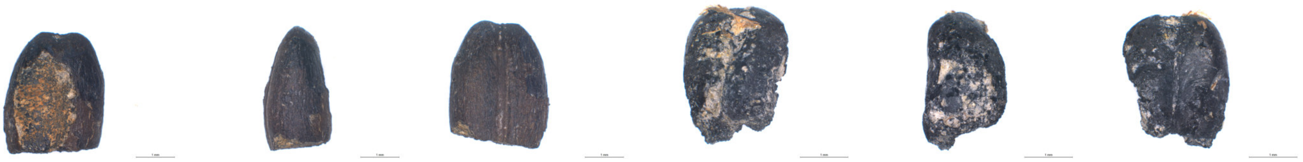

AGRI_C_0552_D AGRI_C_0552_L AGRI_C_0552_V AGRI_C_0553_D AGRI_C_0553_L AGRI_C_0553_V

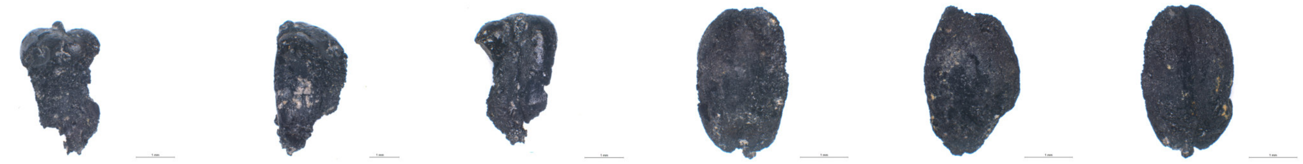

AGRI_C_0554_D AGRI_C_0554_L AGRI_C_0554_V AGRI_C_0555_D AGRI_C_0555_L AGRI_C_0555_V

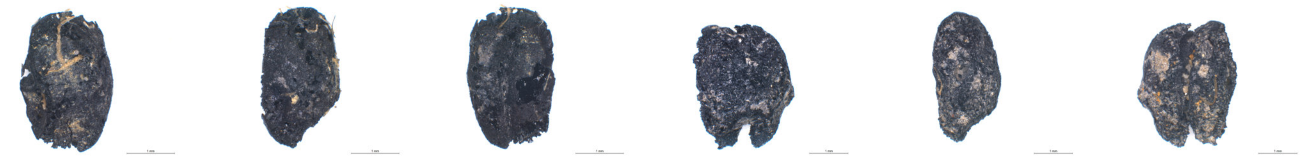

AGRI_C_0556_D AGRI_C_0556_L AGRI_C_0556_V AGRI_C_0557_D AGRI_C_0557_L AGRI_C_0557_V

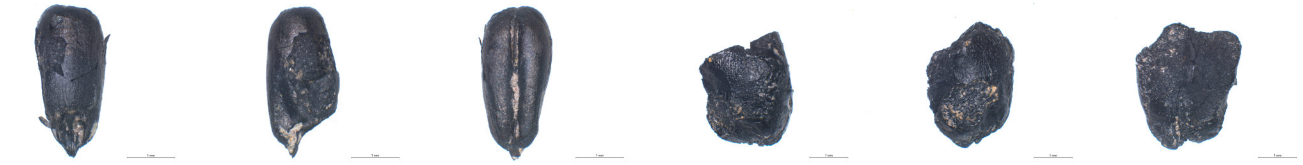

AGRI_C_0558_D AGRI_C_0558_L AGRI_C_0558_V AGRI_C_0559_D AGRI_C_0559_L AGRI_C_0559_V

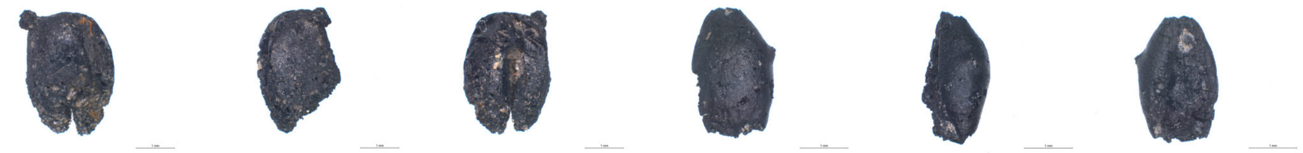

AGRI_C_0560_D AGRI_C_0560_L AGRI_C_0560_V AGRI_C_0561_D AGRI_C_0561_L AGRI_C_0561_V

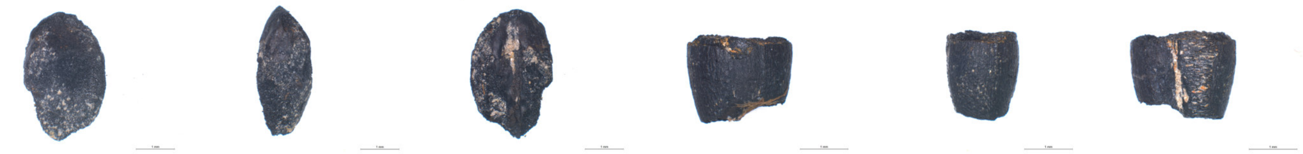

AGRI_C_0562_D AGRI_C_0562_L AGRI_C_0562_V AGRI_C_0563_D AGRI_C_0563_L AGRI_C_0563_V

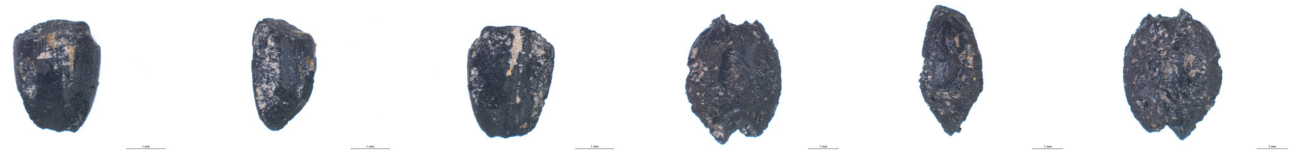

AGRI_C_0564_D AGRI_C_0564_L AGRI_C_0564_V AGRI_C_0565_D AGRI_C_0565_L AGRI_C_0565_V

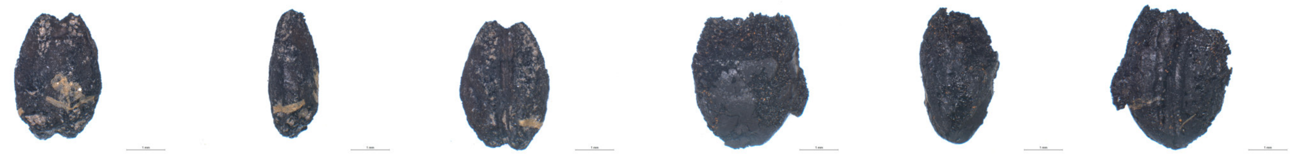

AGRI_C_0566_D AGRI_C_0566_L AGRI_C_0566_V AGRI_C_0567_D AGRI_C_0567_L AGRI_C_0567_V

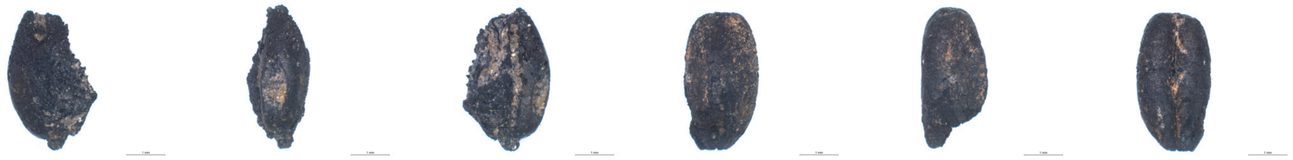

AGRI_C_0568_D AGRI_C_0568_L AGRI_C_0568_V AGRI_C_0569_D AGRI_C_0569_L AGRI_C_0569_V

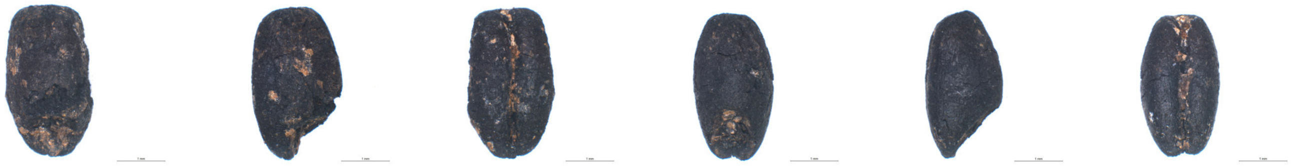

AGRI_C_0570_D.tif AGRI_C_0570_L AGRI_C_0570_V AGRI_C_0571_D AGRI_C_0571_L AGRI_C_0571_V

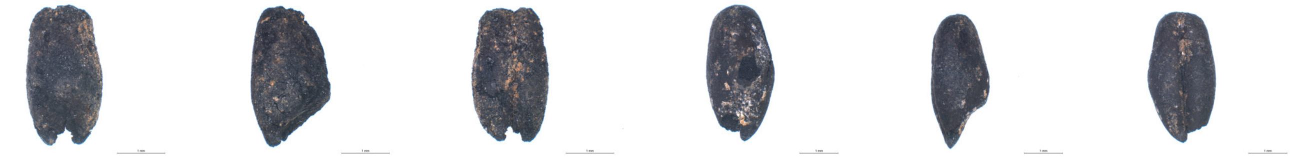

AGRI_C_0572_D AGRI_C_0572_L AGRI_C_0572_V AGRI_C_0573_D AGRI_C_0573_L AGRI_C_0573_V

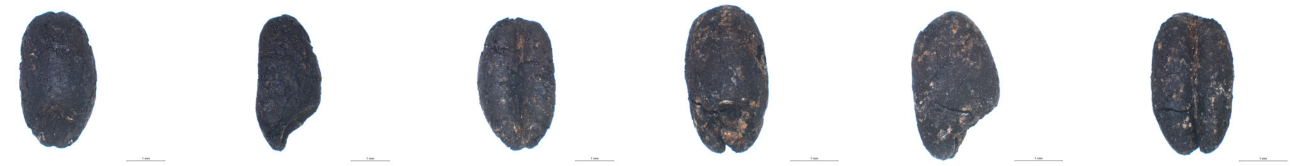

AGRI_C_0574_D AGRI_C_0574_L AGRI_C_0574_V AGRI_C_0575_D AGRI_C_0575_L AGRI_C_0575_V

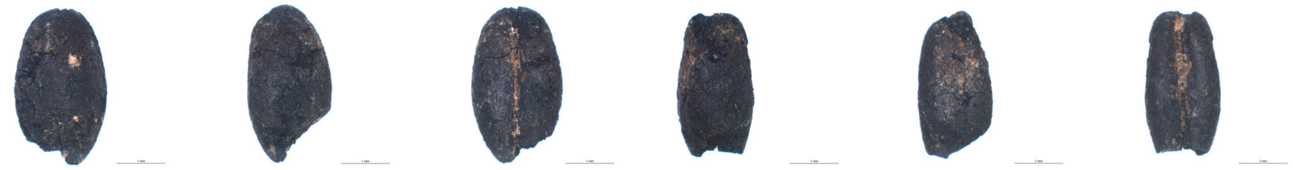

AGRI_C_0576_D AGRI_C_0576_L AGRI_C_0576_V AGRI_C_0577_D AGRI_C_0577_L AGRI_C_0577_V

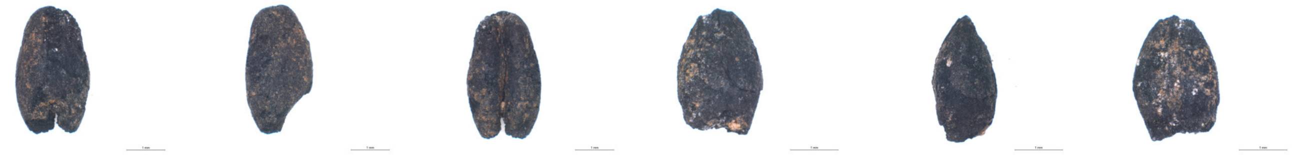

AGRI_C_0578_D AGRI_C_0578_L AGRI_C_0578_V AGRI_C_0579_D AGRI_C_0579_L AGRI_C_0579_V

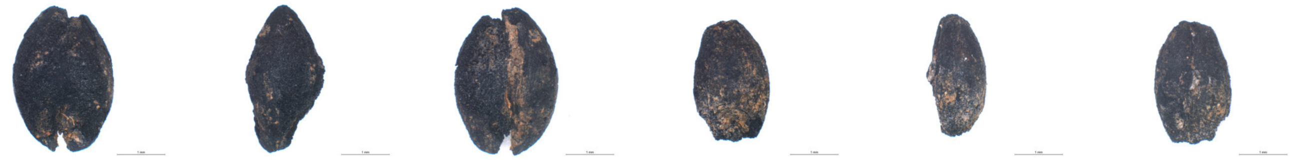

AGRI_C_0580_D AGRI_C_0580_L AGRI_C_0580_V AGRI_C_0581_D AGRI_C_0581_L AGRI_C_0581_V

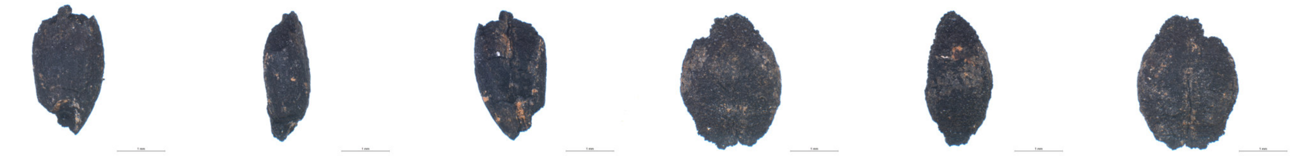

AGRI_C_0582_D AGRI_C_0582_L AGRI_C_0582_V AGRI_C_0583_D AGRI_C_0583_L AGRI_C_0583_V

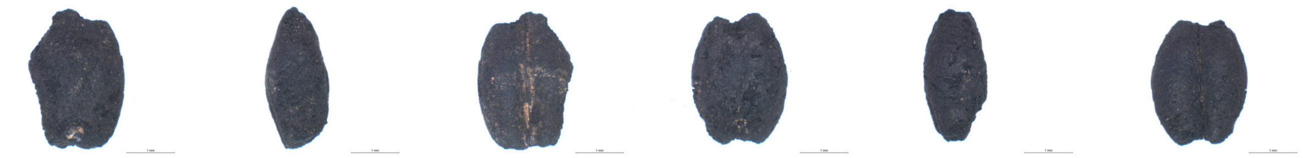

AGRI_C_0584_D AGRI_C_0584_L AGRI_C_0584_V AGRI_C_0585_D AGRI_C_0585_L AGRI_C_0585_V.tif

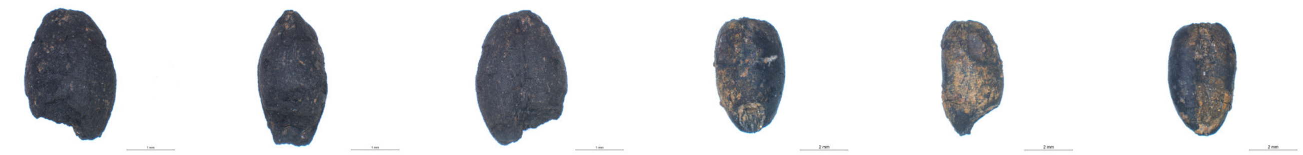

AGRI_C_0586_D AGRI_C_0586_L AGRI_C_0586_V AGRI_C_1168_D AGRI_C_1168_L AGRI_C_1168_V

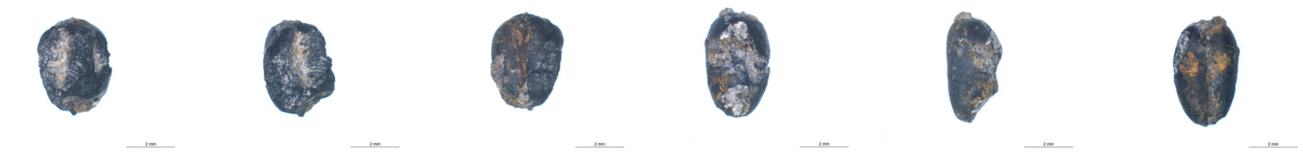

AGRI_C_1169_D AGRI_C_1169_L AGRI_C_1169_V AGRI_C_1170_D AGRI_C_1170_L AGRI_C_1170_V

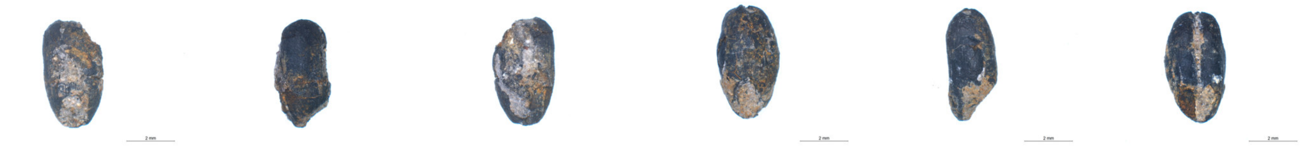

AGRI_C_1171_D AGRI_C_1171_L AGRI_C_1171_V AGRI_C_1172_D AGRI_C_1172_L AGRI_C_1172_V

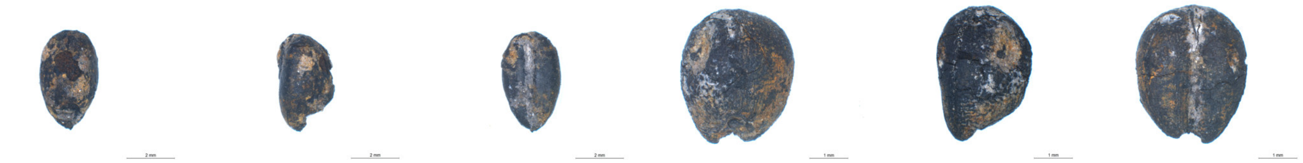

AGRI_C_1173_D AGRI_C_1173_L AGRI_C_1173_V AGRI_C_1174_D AGRI_C_1174_L AGRI_C_1174_V

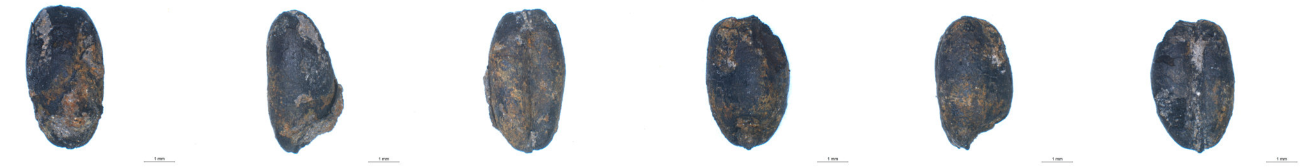

AGRI_C_1175_D AGRI_C_1175_L AGRI_C_1175_V AGRI_C_1176_D AGRI_C_1176_L AGRI_C_1176_V

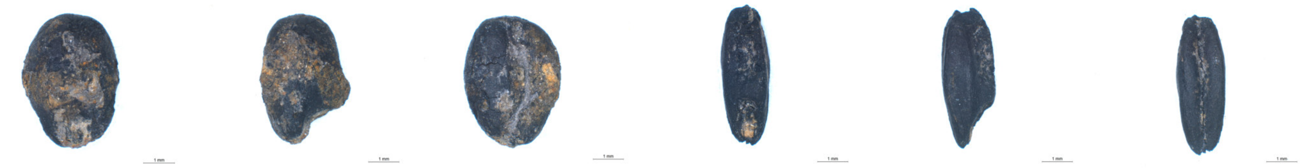

AGRI_C_1177_D AGRI_C_1177_L AGRI_C_1177_V AGRI_C_1178_D AGRI_C_1178_L AGRI_C_1178_V

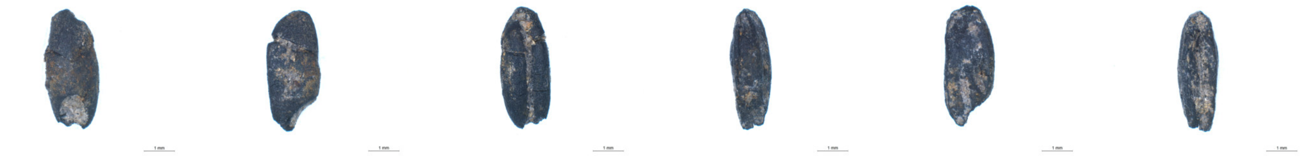

AGRI_C_1179_D AGRI_C_1179_L AGRI_C_1179_V AGRI_C_1180_D AGRI_C_1180_L AGRI_C_1180_V

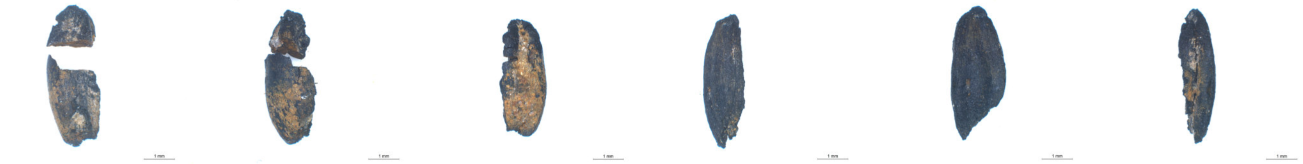

AGRI_C_1181_D AGRI_C_1181_L AGRI_C_1181_V AGRI_C_1182_D AGRI_C_1182_L AGRI_C_1182_V

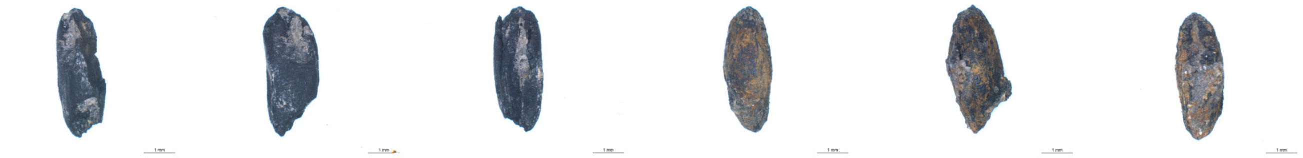

AGRI_C_1183_D AGRI_C_1183_L AGRI_C_1183_V AGRI_C_1184_D AGRI_C_1184_L AGRI_C_1184_V

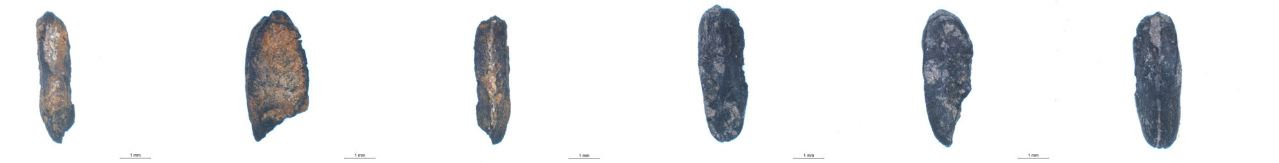

AGRI_C_1185_D AGRI_C_1185_L AGRI_C_1185_V AGRI_C_1186_D AGRI_C_1186_L AGRI_C_1186_V

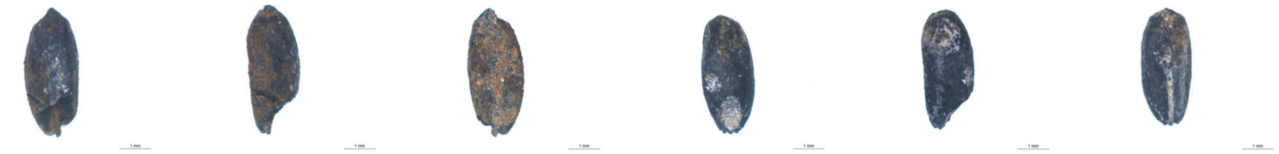

AGRI_C_1187_D AGRI_C_1187_L AGRI_C_1187_V AGRI_C_1188_D AGRI_C_1188_L AGRI_C_1188_V

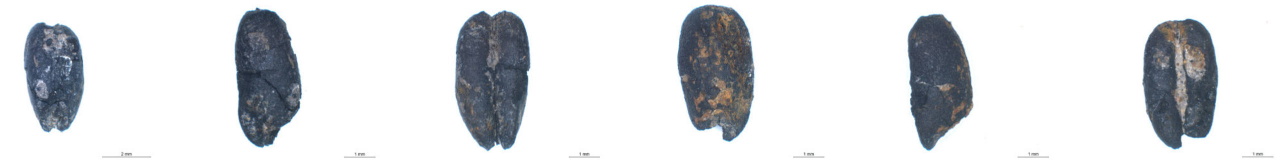

AGRI_C_1189_D AGRI_C_1189_L AGRI_C_1189_V AGRI_C_1190_D AGRI_C_1190_L AGRI_C_1190_V

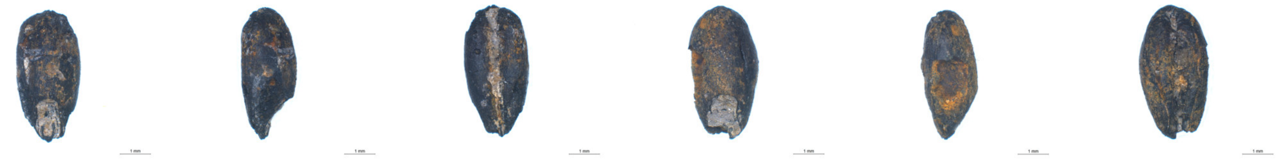

AGRI_C_1191_D AGRI_C_1191_L AGRI_C_1191_V AGRI_C_1192_D AGRI_C_1192_L AGRI_C_1192_V

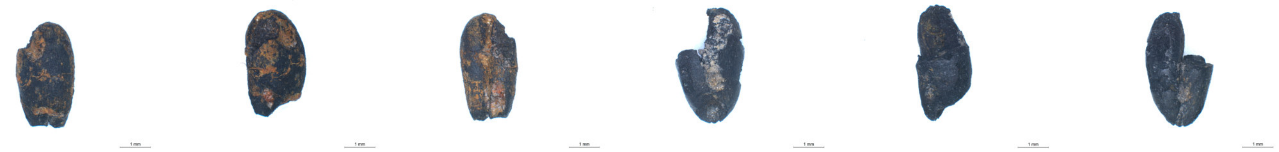

AGRI_C_1193_D AGRI_C_1193_L AGRI_C_1193_V AGRI_C_1194_D AGRI_C_1194_L AGRI_C_1194_V

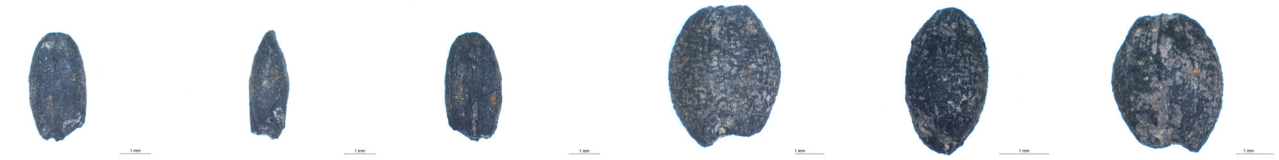

AGRI_C_1195_D AGRI_C_1195_L AGRI_C_1195_V AGRI_C_1196_D AGRI_C_1196_L AGRI_C_1196_V

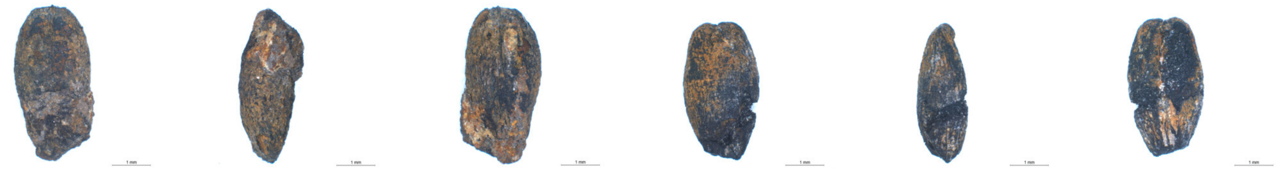

AGRI_C_1197_D AGRI_C_1197_L AGRI_C_1197_V AGRI_C_1198_D AGRI_C_1198_L AGRI_C_1198_V

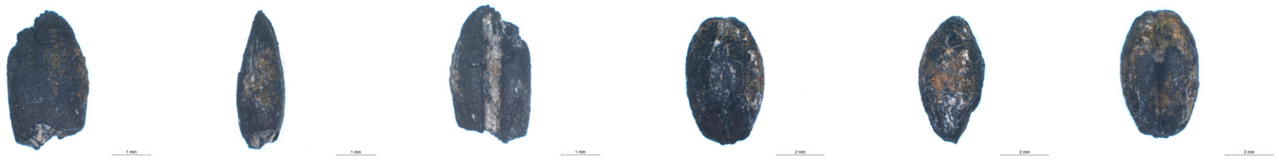

AGRI_C_1199_D AGRI_C_1199_L AGRI_C_1199_V AGRI_C_1200_D AGRI_C_1200_L AGRI_C_1200_V

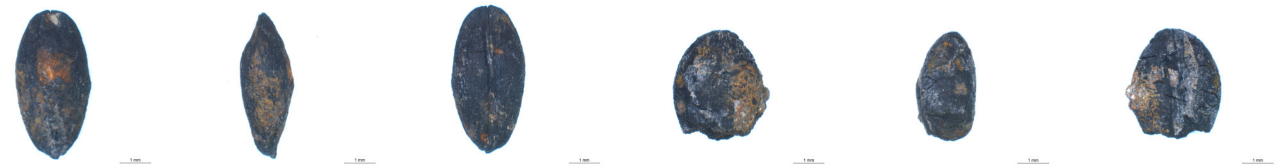

AGRI_C_1201_D AGRI_C_1201_L AGRI_C_1201_V AGRI_C_1202_D AGRI_C_1202_L AGRI_C_1202_V

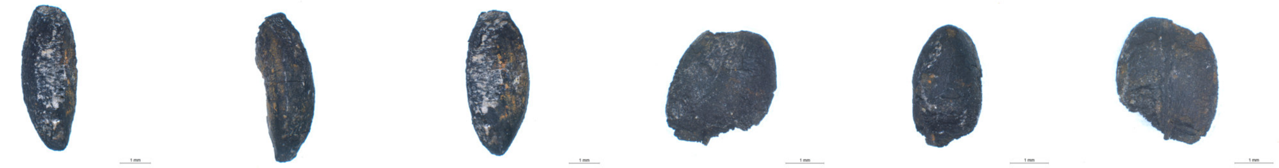

AGRI_C_1203_D AGRI_C_1203_L AGRI_C_1203_V AGRI_C_1204_D AGRI_C_1204_L AGRI_C_1204_V
