# Supplementary material for: An archaeobotanical and stable isotope approach to changing agricultural practices in the NW Mediterranean region around 4000 BC
Source: Holocene. 2023 Dec 4;34(2):239–54. doi: 10.1177/09596836231211848 (PMC10799764; doi:10.1177/09596836231211848)
Supplement: Supplementary file 3 [file sj-docx-4-hol-10.1177_09596836231211848.docx]

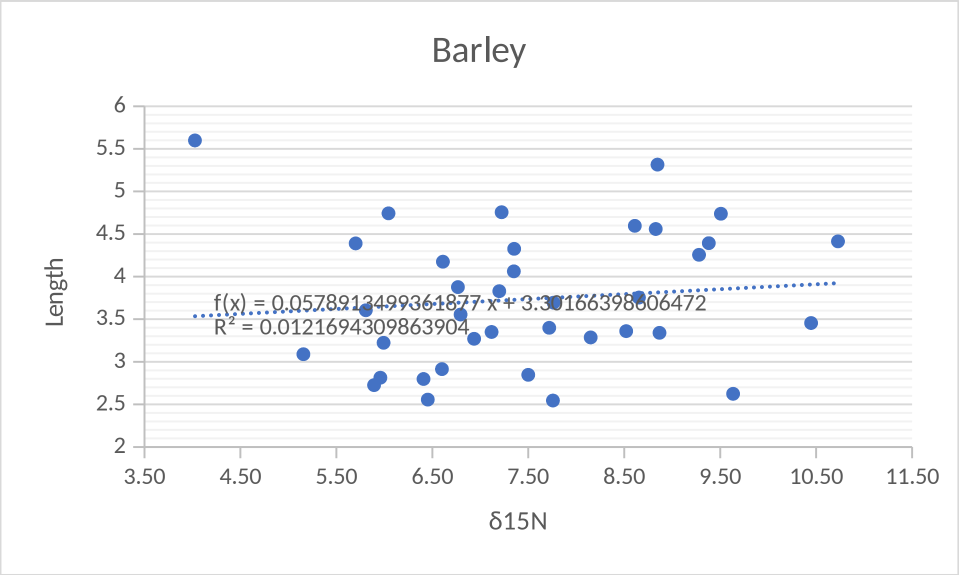
Barley
6
5.5
5
4.5
4
3.5
3
2.5
2
f(x) = 0.0578913499361877 x + 3.30166398806472
R² = 0.0121694309863904
3.50
4.50
5.50
6.50
7.50
8.50
9.50
10.50
11.50
δ15N
Length

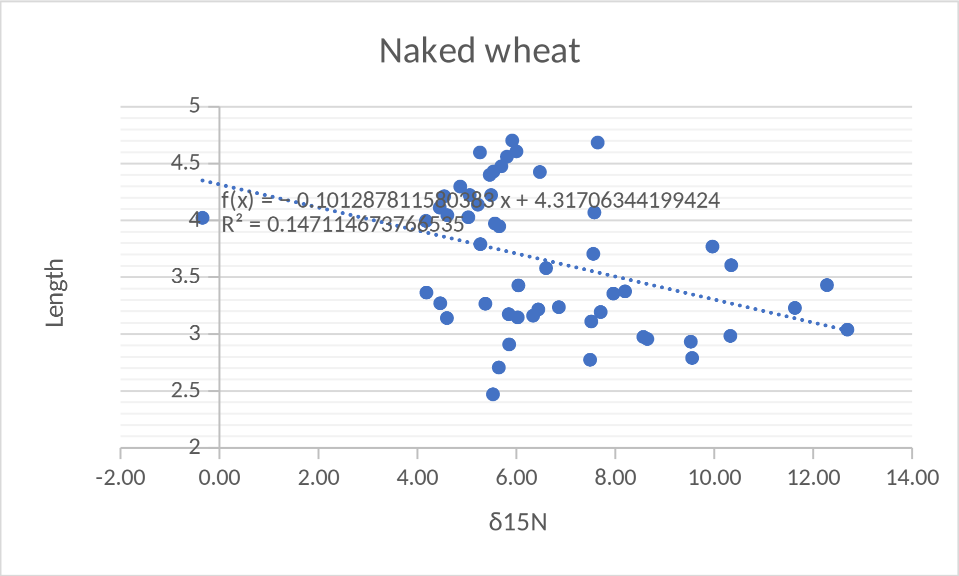
Naked wheat
5
4.5
4
3.5
3
2.5
2
f(x) = − 0.101287811580383 x + 4.31706344199424
R² = 0.147114673766535
-2.00
0.00
2.00
4.00
6.00
8.00
10.00
12.00
14.00
δ15N
Length

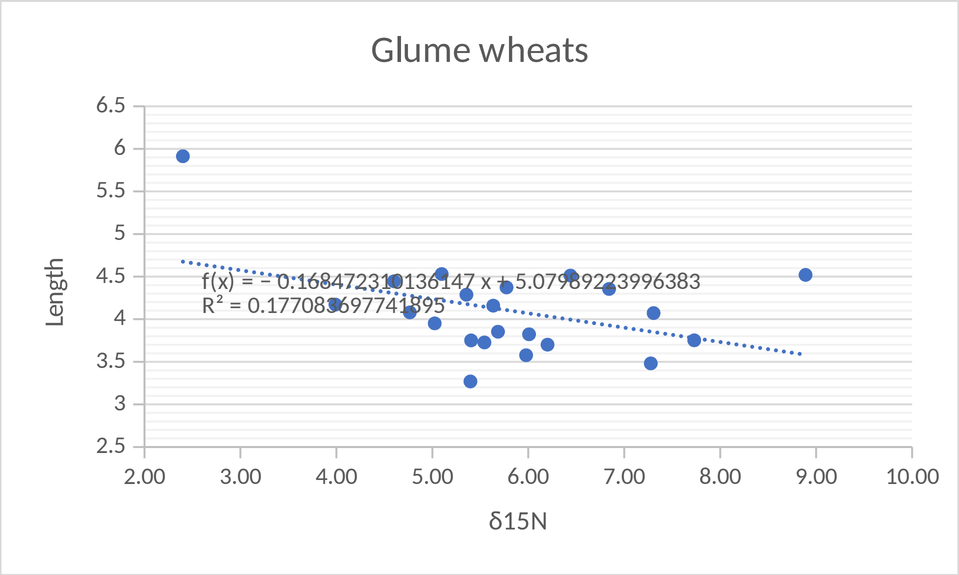

Glume wheats
6.5
6
5.5
5
4.5
4
3.5
3
2.5
Length
f(x) = − 0.168472310136147 x + 5.07969223996383
R² = 0.177083697741895
2.00
3.00
4.00
5.00
6.00
7.00
8.00
9.00
10.00
δ15N
