# Supplementary material for: An archaeobotanical and stable isotope approach to changing agricultural practices in the NW Mediterranean region around 4000 BC
Source: Holocene. 2023 Dec 4;34(2):239–54. doi: 10.1177/09596836231211848 (PMC10799764; doi:10.1177/09596836231211848)
Supplement: Supplementary file 4 [file sj-docx-5-hol-10.1177_09596836231211848.docx]

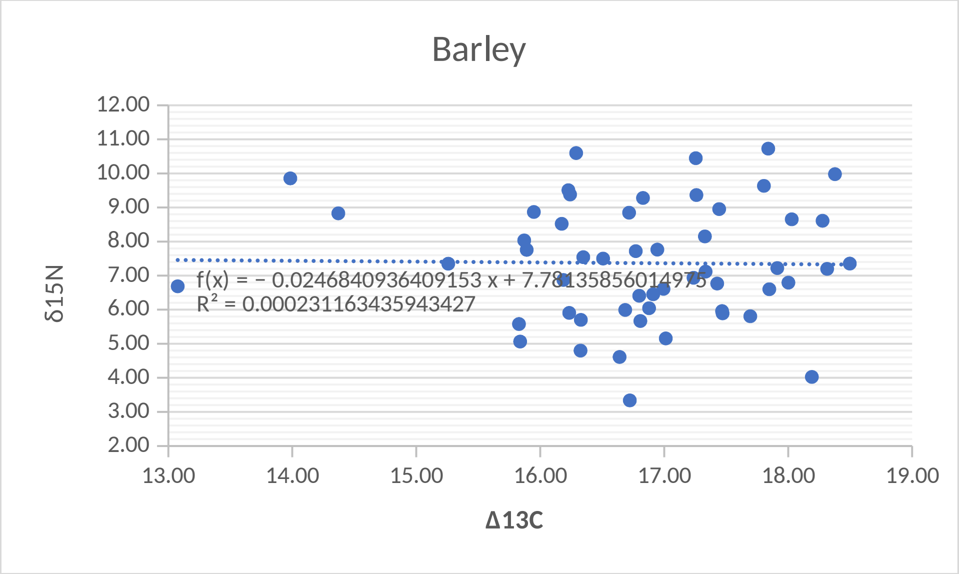
Barley
12.00
11.00
10.00
9.00
8.00
7.00
6.00
5.00
4.00
3.00
2.00
δ15N
f(x) = − 0.0246840936409153 x + 7.78135856014975
R² = 0.000231163435943427
13.00
14.00
15.00
16.00
17.00
18.00
19.00
Δ13C

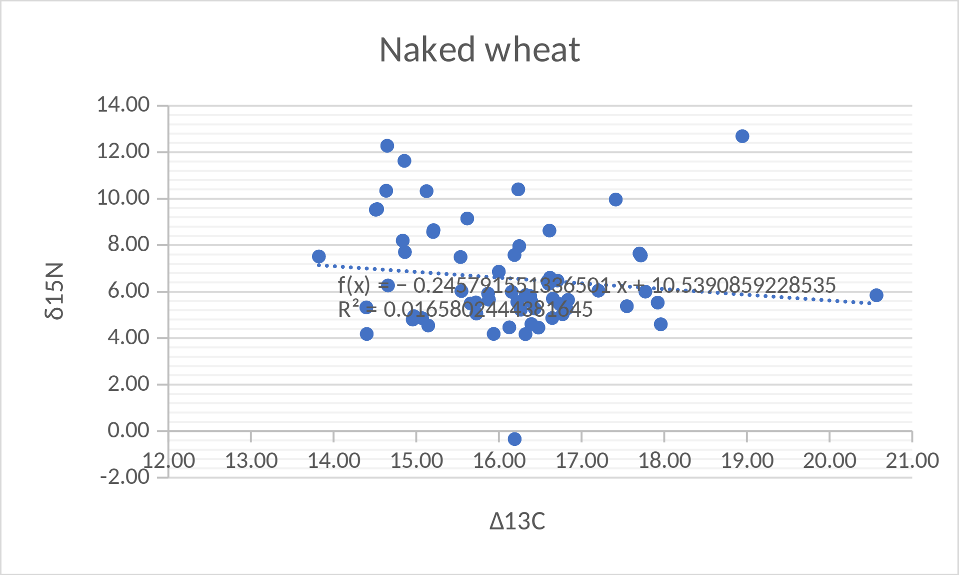
Naked wheat
14.00
12.00
10.00
8.00
6.00
4.00
2.00
0.00
-2.00
δ15N
f(x) = − 0.245791551336501 x + 10.5390859228535
R² = 0.0165802444381645
12.00
13.00
14.00
15.00
16.00
17.00
18.00
19.00
20.00
21.00
Δ13C

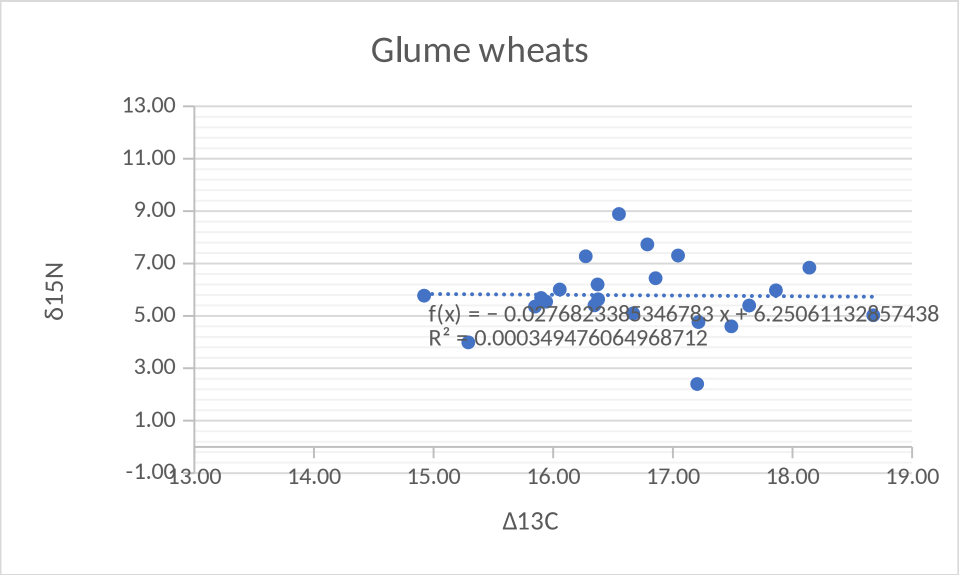

Glume wheats
13.00
11.00
9.00
7.00
5.00
3.00
1.00
-1.00
δ15N
f(x) = − 0.0276823385346783 x + 6.25061132857438
R² = 0.000349476064968712
13.00
14.00
15.00
16.00
17.00
18.00
19.00
Δ13C
